# Medium throughput breathing human primary cell alveolus-on-chip model

**DOI:** 10.1038/s41598-018-32523-x

**Published:** 2018-09-25

**Authors:** Janick D. Stucki, Nina Hobi, Artur Galimov, Andreas O. Stucki, Nicole Schneider-Daum, Claus-Michael Lehr, Hanno Huwer, Manfred Frick, Manuela Funke-Chambour, Thomas Geiser, Olivier T. Guenat

**Affiliations:** 1 ARTORG Center for Biomedical Engineering Research, Organs-on-Chip Technologies, University of Bern, Bern, Switzerland; 2 Institute of General Physiology, University of Ulm, Ulm, Germany; 3 Helmholtz Institute for Pharmaceutical Research Saarland, Helmholtz Center for Infection Research, Biopharmaceutics and Pharmaceutical Technology, Saarland University, Saarbrücken, Germany; 4 SHG Clinics, Department of Cardiothoracic Surgery, Völklingen Heart Center, Völklingen, Germany; 5 Department of Pulmonary Medicine, University Hospital of Bern, Bern, Switzerland; 6 Department for Biomedical Research, University of Bern, Bern, Switzerland; 7 Division of General Thoracic Surgery, University Hospital of Bern, Bern, Switzerland; 8 AlveoliX AG, Bern, Switzerland

## Abstract

Organs-on-chips have the potential to improve drug development efficiency and decrease the need for animal testing. For the successful integration of these devices in research and industry, they must reproduce *in vivo* contexts as closely as possible and be easy to use. Here, we describe a ‘breathing’ lung-on-chip array equipped with a passive medium exchange mechanism that provide an *in vivo*-like environment to primary human lung alveolar cells (hAEpCs) and primary lung endothelial cells. This configuration allows the preservation of the phenotype and the function of hAEpCs for several days, the conservation of the epithelial barrier functionality, while enabling simple sampling of the supernatant from the basal chamber. In addition, the chip design increases experimental throughput and enables trans-epithelial electrical resistance measurements using standard equipment. Biological validation revealed that human primary alveolar type I (ATI) and type II-like (ATII) epithelial cells could be successfully cultured on the chip over multiple days. Moreover, the effect of the physiological cyclic strain showed that the epithelial barrier permeability was significantly affected. Long-term co-culture of primary human lung epithelial and endothelial cells demonstrated the potential of the lung-on-chip array for reproducible cell culture under physiological conditions. Thus, this breathing lung-on-chip array, in combination with patients’ primary ATI, ATII, and lung endothelial cells, has the potential to become a valuable tool for lung research, drug discovery and precision medicine.

## Introduction

Organs-on-chips are advanced *in vitro* models mimicking the cellular microenvironment found *in vivo*^1^. These models are widely seen as promising tools for preclinical validation and have the potential to improve and accelerate drug development^2–4^. However, to make organs-on-chip widely available, key challenges must be overcome. First, these systems must mimic the cellular microenvironment. In addition, it is necessary to consider handling issues, such as ease of access to the cells, the potential of long-term culture and analysis of cells, and simplicity of handling^5^. In particular, long-term experiments are crucial for observation of cell–cell interactions that occur over extended time periods (e.g., cellular differentiation or pathological conditions such as fibrosis)^4^. Such experimental designs require regular medium exchange to provide sufficient nutrients and remove metabolic waste products. Previous organ-on-chip studies report medium exchange by active or passive pumping systems, either external or directly integrated on-chip^6–11^. Active pumping is often cumbersome, due to the requirement for tubing and external equipment, but passive pumping offers simple and elegant solutions. Passive systems can be based on surface tension^12^, hydrostatic^13–15^, capillary forces^16^ or evaporation effects^17,18^. Moreover, because they do not contain any moving parts, they are simple to produce and mechanically robust.

Due to their complex architecture located in a dynamic environment, the lung alveoli are difficult to mimic *in-vitro*. *In vivo*, the air–blood interface is an ultrathin barrier of only a few micrometers^19^, consisting of tight, semi-selective epithelial- and endothelial cell layers enveloped by the extracellular matrix (ECM)^20,21^, rhythmically expanding and contracting. Numerous 2D and 3D *in-vitro* models of the lung alveolar barrier have been reported^22,23^, but only few reproduced the mechanical forces created by the respiratory movements. In 2007, Takayama and colleagues mimicked those forces using liquid plugs transported by airflow to assess cellular damage^24^. In a further study, they investigated the effects of physiological and pathophysiological levels of both fluid and solid mechanical stresses on alveolar epithelial cells^25^. Using a similar technique, Higuita *et al*. investigated how airway wall stiffness influences epithelial cell injury^26^. More recently, Schürch *et al*. used a captive bubble surfactometer system^27^, to expose the surfactant film lining the epithelium to cyclic compression and expansion^28^. In addition to the cyclic stretch, Campillo *et al*. developed an *in-vitro* system equally capable of inducing high frequency intermittent hypoxia, a hallmark of obstructive sleep apnea^29^.

In sharp contrast to the *in-vitro* models mentioned above, Huh *et al*. reported in 2010 for the first time about a microfluidic system with an integrated lung alveolar barrier that was cyclically stretched in one direction to mimic the respiratory movements. The device, called lung-on-chip, was equipped with a thin, porous and elastic membrane, on which alveolar epithelial cells and endothelial cells were cultured on the apical and basal side, respectively^6^. More recently our group reported about a new lung-on-chip device with an alveolar barrier stretched in three dimensions, similar to the *in vivo* situation. The breathing movements were induced by the cyclic deflection of a microdiaphragm, similar to the movements of the *in-vivo* diaphragm. Primary human lung alveolar epithelial cells from patients were cultured under physiological mechanical strain for the first time on such a device^30^. Lately, Jain *et al*. described an alveolus-on-a-chip model using primary human lung alveolar epithelial and lung endothelial cells to mimic pulmonary thrombosis. In the later case, the cells were however not exposed to cyclic strain^31^.

Here, we present a new lung-on-chip device equipped with a pumping system that combines both active and passive pumping, enabling passive medium flow at freely chosen time points. In contrast to our earlier work, the new lung-on-chip is equipped with passive medium exchange that enables not only the reproduction of the cyclic mechanical stress, but also long-term cell culture at the air–liquid interface, and thus reproduces the unique aspects of the lung microenvironment even more closely. The details of the microenvironment have significant impacts on a wide range of biological processes, including epithelial cell polarity and cell differentiation, which in turn play important roles in determining physiological functions^32–38^. Furthermore, we describe a breathing lung alveolar barrier consisting of primary human alveolar epithelial and lung endothelial cells cultured in an *in vivo*–like environment. The presence of type I- (ATI) and type II-like (ATII) alveolar epithelial phenotypes is demonstrated for the first time in a lung-on-chip. Using this system, we assessed the effect of physiological stretching on cell morphology and barrier permeability of primary ATI- and ATII-like cells. Importantly, this new system is compatible with a number of standard readout techniques, including ELISA, trans-epithelial electrical resistance (TEER), permeability assays, qPCR, immunostaining and electron microscopy. This device opens up new possibilities for basic research of the alveoli and preclinical testing of drug candidates, and will ultimately enable testing of cells from individual patients in precision medicine approaches.

## Materials and Methods

### Lung-on-chip design and fabrication

A detailed description of the design and of the operation of the lung-on-chip is given in the supplementary section (see supplementary information “Lung-on-Chip design and operation”). Briefly, the lung-on-chip consists of reversibly bonded fluidic and pneumatic parts with six independent lung alveolar barriers (Fig. S1). The fluidic part comprises two structured plates – the top and middle plates – between which a thin, porous, elastic PDMS membrane is sandwiched. The top plate is made of either PDMS or polycarbonate (PC), whereas the middle plate is made of PDMS. The pneumatic part comprises a structured PDMS base plate, on which a 40-µm thick PDMS membrane is attached by plasma O_2_ (Harrick plasma). The top and PDMS middle plate of the fluidic part, and the base plate of the pneumatic part were produced by soft lithography^39^. Briefly, 10:1 PDMS (Sylgard 184, Dow Corning) was mixed, degassed and casted in hard plastic molds (Weidling C, Weicon) obtained from structured aluminum molds (ki-Mech GmbH). The PDMS was cured in an oven at 60 °C for at least 24 h. The 3 mm in diameter elastic membrane with pores of 3-µm (density of the pores: 800’000 pores/cm^2^) was fabricated as described previously^30^. In brief, 10:1 PDMS pre-polymer was spread between a silicon mold and a PET sheet (DuPont Teijin Films, Melinex® 411) using a roller. The mold and PET sheet were then clamped between two glass slides and cured at 60 °C for at least 24 h. Afterwards, the membrane was released and bonded to the middle plate with O_2_ plasma (Harrick Plasma). The PC top plate, fabricated by standard machining, was first coated with 25 μL of Wacker PRIMER G790 (Ameba AG) and placed on a hot plate (80 °C for 20 min). Then, the PC top was placed on an uncured 40-µm layer of PDMS– spin coated on a glass plate (1700 rpm for 60 s) – cured overnight in the oven at 60 °C and removed from the glass plate. The middle part with the bonded thin and porous membrane was then assembled with the PDMS coated top part. To assemble the lung-on-chip array, the pneumatic and fluidic parts were visually aligned, brought in contact, and reversibly attached by manual pressing using adhesion forces alone.

### Lung-on-chip validation

#### Initial chip filling

The lung-on-chip was assembled, and the inlet well was filled with cell culture medium. To fill the chip with medium, the valves were opened, and negative pressure was applied at the outlet well using a 1 mL syringe. Filling was recorded with a color camera (iDS UI-3370CP-C-HQ, iDS).

#### Medium exchange

The lung-on-chip was assembled, filled with cell culture medium, and placed in an incubator for at least 2 h. The inlet and outlet wells were emptied, the inlet well was filled with fluorescein sodium (Sigma-Aldrich) in PBS (1 mg mL^−1^), and the valves were opened to initiate the medium exchange. Medium exchange was monitored in fluorescence mode with an upright microscope (Axioplan 2, Zeiss) equipped with a color camera (iDS UI-3370CP-C-HQ, iDS). The resultant movie was processed using the open-source image analysis software Fiji (http://fiji.sc/Fiji). After cropping to the correct starting time, corresponding to the time point when the valves opened, a kymograph (pixel intensity along a defined line over time) was created of the beginning of the outlet channel of the basal cell culture chamber. The kymograph was used to determine the time at which the intensity signal no longer changed, which corresponds to complete filling of the basal chamber (i.e., the end of the exchange).

#### Thin membrane deflection

As described above, the chip was assembled, filled with cell culture medium, and placed in the incubator overnight. Membrane deflection was monitored with an Axioplan 2 upright microscope equipped with a high-speed camera (Basler piA640-210gc GigE, Basler AG). The inlet and outlet wells were emptied completely, and then the inlet well was filled with cell culture medium. During the exchange, a small light spot was projected onto the membrane that changed its shape according to the deflection. The movement of the membrane (i.e., the shape change of the light spot) was recorded at 200 fps. The movie was then processed using Fiji. Brightness and contrast were adjusted, and kymograph was created for a line through the center of the light spot. Afterwards, the edges were detected, and the data (x- and y-positions) of the edges were saved. The data were then further analyzed in Matlab (MathWorks) to extract the corresponding deflection. For more information, see Fig. S2.

#### Applied strain

The lung-on-chip was assembled and filled with cell culture medium. The mechanical strain generated in the device was quantified by taking images in the center of the membrane in the non-deflected and deflected states. These images were processed in Fiji and Matlab to calculate the inter-pore distance, which was then used to compute the linear strain (Fig. S3).

### Standard cell culture protocols

Bronchial epithelial 16HBE14o- cells (obtained from the late Dr. Dieter Gruenert, University of California–San Francisco) were cultured in MEM medium (Gibco) supplemented with 10% FBS (Gibco), 1% L-Glutamine (2 mM, Gibco), 1% penicillin, and 1% streptomycin (P/S, Gibco). RFP labeled primary human lung microvascular endothelial cells (VeraVec, Angiocrine Bioscience) were cultured in EGM™−2 medium (Lonza) supplemented with 5% FBS (Sigma), 1% penicillin (Gibco), 1% streptomycin (Gibco), and VEGF growth factors according to the manufacturers protocol for the EGM™-2MV BulletKit. Primary human alveolar epithelial cells (hAEpCs) were isolated according to an established protocol described by Daum *et al*.^40^. Briefly, alveolar epithelial type II (AT II) cells were isolated from tissue obtained from healthy areas removed from patients undergoing lung tumor resection surgery. All patients gave informed written consent for usage of surgical material for research purposes, which was approved by ethical committee from the Ärztekammer des Saarlandes. All procedures were carried out in accordance with institutional guidelines from Saarland (Germany) and from the Canton of Bern (Switzerland). The ATII cell population was purified by a combination of cell attachment procedure, density gradient centrifugation via Percoll®, and positive magnetic cell sorting as in detailed described in Daum *et al*.^40^. Directly after isolation, hAEpCs were resuspended in 40 mL Small Airway Growth Medium (SAGM™, Lonza) with BulletKit (CC-3118, Lonza), supplemented with 1% FBS (Sigma) and 1% P/S. All cell types were maintained in a standard cell culture environment (37 °C, 5% CO_2_ in air). To verify the integrity and barrier function of shipped hAEpCs, primary cells were seeded on 0.33 cm^2^ Transwell® filters (Corning, C#3470), and TEER was measured daily with a chopstick electrode connected to an EVOM2 epithelial volt/ohm meter (World Precision Instruments) as previously described^40^ (data not shown).

### Coating and cell seeding on chip

When using the 16HBE14o- cell line, the porous PDMS membrane was coated with collagen IV (2.5 µg cm^−2^, Sigma-Aldrich) overnight in the incubator. The cells were seeded at a density of 1.5 × 10^5^ cells cm^−2^ in a 15 µL droplet on the apical side of the membrane. After adhesion (2 h), the apical cell culture well was filled to 80 µL. When working with primary cells, the porous PDMS membrane was coated with a bovine collagen I (Sigma-Aldrich) and human fibronectin (Corning) solution, for 4 hours in the incubator, as previously described for inserts by Daum *et al*.^40^. The coating solution was removed, and the membrane rinsed with 80 µL of sterile water. The chips were then placed under UV for 45 min to allow drying. For hAEpC mono-culture, the chips were first closed and filled with cell culture medium. Then, the hAEpCs were seeded (3 × 10^5^ cells cm^−2^) on the apical side of the membrane in a 15 µL droplet. After 4 h, the cell culture well was filled to 80 µL with cell culture medium. In co-culture experiments, VeraVec cells (3 × 10^4^ cells cm^−2^) were first seeded – using a standard pipette – on the basal side of the membrane in a 15 µL droplet. For this procedure, the fluidic part of the chip was flipped so that the basal side of the porous membrane faces upwards. After 4 h, once the endothelial cells adhered on the membrane, 5 µL were removed from the drop, and the fluidic part flipped back. The fluidic part was then assembled to the pneumatic part, to close the chip, which was then filled with medium, see Fig. S2. Afterwards, the hAEpCs (3 × 10^5^ cells cm^−2^) were seeded on the apical side in a 15 µL droplet. After 4 h, the cell culture well was filled to 80 µL with cell culture medium.

### Cell viability assay

16HBE14o- cells were grown in MEM with 10% FBS until they reached confluence (3 days, daily medium exchange). The apical supernatant was collected, and the chip was closed and filled with MEM with 1% FBS (MEMs). Cell viability was measured using the PrestoBlue® assay (Life Technologies). In brief, after 1 h incubation with PrestoBlue® (1:10 in MEMs), 50 μL of apical solution was collected and transferred to a 96-well plate. The readout was carried out on a multi-well plate reader (M1000 Infinite, Tecan) at 570 nm excitation and 585 nm emission. The apical chamber was washed with PBS with calcium, and the medium in the apical and basal cell culture chamber was replaced with MEMs. The chips were then transferred to an incubator and connected to the external electro-pneumatic setup. They were either stretched (dynamic) or maintained under static conditions. Viability was measured daily in the supernatant from the apical side.

### Long-term alveolar epithelial and endothelial co-culture

As described above, the VeraVec (P. 2) cells were seeded on the basal side. After cell attachment, the chip was closed and filled with EGM™-MV2. VeraVec cells were grown on the chip for 2 days (with no medium on the apical side) in EGM™-2MV. Then, the EGM™-2MV medium was exchanged with a 1:1 mixture of EGM™-2MV and SAGM™. After medium exchange, the hAEpCs were seeded on the apical side, as described above (day 0). Cell culture medium was exchanged every second day. TEER measurements started at day 1. TEER was measured daily using a commercially available 96-well plate electrode (STX100M; World Precision Instruments) and an EVOM2. The TEER was measured in submerged cell culture conditions for up to 22 days. The specific design of the lung-on-chip enables to hold the electrodes tightly between the outlet well and the culture well. This allows an accurate and reproducible positioning of the TEER electrodes. To measure TEER, electrodes were placed into the cell culture well and outlet well, and then the valves were opened. TEER background was measured on a porous membrane chip containing no cells. Background-subtracted TEER values (Ω) were multiplied by the surface area (0.07 cm^2^) to calculate Ω × cm^2^.

### Stretching protocol

The lung-on-chip was designed to create a three-dimensional physiological surface strain of 16.6%. In view to make cross-comparison^41,42^ possible with other systems using a uniaxial strain, the surface strain is indicated in the following as linear strain of 8%. The membrane was cyclically stretched at 0.2 Hz using an external electro-pneumatic setup. The pressure curve was modeled as a triangle wave.

### qRT-PCR protocol

Total RNA was isolated and purified from genomic DNA using the NucleoSpin® RNA XS kit (Macherey Nagel). To harvest cells from the lung-on-chip, an aliquot of 100 μL lysis buffer was applied to every well, pipetted up and down three times, and transferred to a separate tube. RNA concentration and purity was analyzed on a NanoDrop Lite (Thermo Scientific). Purified total RNA was used for cDNA preparation with the Super Script III Reverse Transcriptase kit (Life Technologies) employing random hexamer primers. cDNAs were amplified by quantitative real-time PCR using SYBR® Select Master Mix (Thermo Scientific) on a 7500 Fast Start Real-Time PCR system (Applied Biosystems). Primers for ENACα (Cat.no: QT0002883) and Caveolin1 (Cat.no: QT00012607) were purchased from Qiagen. The primer sequences for 18S and hSP-C are provided in Table S1.

### Permeability assay

hAEpCs were seeded on a chip as described above and cultured on the apical side for 2 days under static and submerged conditions. They were then placed at the air–liquid interface and cultured under static or dynamic conditions for 3 additional days. On day 5, the apical side was washed with PBS with calcium, which was subsequently exchanged with 80 μL of a permeability solution [1 µg mL^−1^ FITC-sodium (0.4 kDa, Sigma-Aldrich) and 1.5 mg mL^−1^ RITC-dextran (70 kDa, Sigma-Aldrich) in SAGM™ medium]. The chip was then transferred to the incubator and incubated in static conditions. After 2 h, the apical permeability solution was removed, the apical side was washed once with PBS with calcium, following which the PBS was removed, and the medium in the basal chamber exchanged. The supernatant solution was collected from the outlet well. A 50-µL aliquot of this solution was transferred to a 96-well flat-bottom plate and analyzed with a microplate reader (M1000 Infinite, Tecan) at 460 nm excitation/515 nm emission for FITC-sodium and 553 nm excitation/627 nm for RITC-dextran. The apparent permeability coefficient (Papp) was calculated according to equation (1), dQ/dt being the transport rate and C_0_, the initial concentration of the permeability solution tested and A the surface area of the permeability barrier.

1 $${P}_{app}=(\frac{dQ}{dt})\,\ast \,\frac{1}{{C}_{0}}\,\ast \,\frac{1}{A}$$

### Immunofluorescence staining and imaging

For immunofluorescence imaging, cells were washed twice with PBS with calcium and fixed for 12 min at room temperature (RT) with 4% paraformaldehyde in PBS without calcium. The fluidic and pneumatic parts were disassembled to allow efficient rinsing. After three washes with PBS without calcium, the cells were permeabilized and blocked for 30 min at RT with 0.1% Triton X-100 in 2% BSA/PBS solution. After an additional wash with PBS without calcium, the samples were ready for staining. All secondary antibodies were purchased from Thermo Fisher Scientific. Mono-culture staining: primary mouse anti-Zo-1 antibody (C# 33-9100, Fisher T Scientific), primary rabbit anti-mature SP-C antibody (C# WRAB-76694, Sevenhills), primary mouse anti-ABCa3 (C# ab24751, Abcam) and primary goat anti-caveolin-1 antibody (C# ab36152, Abcam) were diluted 1:100 in 0.2% BSA and incubated for 1.5 h at RT. After four washes times with PBS, secondary antibody donkey anti-mouse Alexa Fluor 674, donkey anti-rabbit Alexa Fluor 488 (both diluted 1:500) and 1 mg mL^−1^ Hoechst 3342 (diluted 1:1000) in 0.2% BSA/PBS were added, and the sample was incubated for 1 h at RT in the dark. Co-culture staining: Primary anti-E-cadherin goat antibody (C#N-20, Santa Cruz Biotechnology) and Zo-1 diluted 1:100 in 0.2% BSA/PBS were added, and the sample was and incubated for 1.5 h at RT. After four washes times with PBS on both sides, secondary anti-mouse Alexa Fluor 647 and anti-goat Alexa Fluor 488 (1:500) and 1 mg mL^−1^ Hoechst 33342 (1:1000) in 2% BSA/PBS were added, and the sample was incubated for 1.5 h at RT in the dark.

Images were obtained using either a confocal laser scanning microscope (Zeiss LSM 710) or an inverse microscope equipped with a spinning disc (Nikon Ti-E, X-light V2, CrestOptics), using appropriate filter settings. Acquired images were further processed in Fiji to optimize visualization (background subtraction, contrast enhancement, change of color channels, etc.).

### Electron microscopy

After removal of culture media, cells on the membrane were submerged in a fixative, prepared as following: 2.5% glutaraldehyde (Agar Scientific) in 0.15 M HEPES (Fluka) with an osmolarity of 670 mOsm, adjusted to pH 7.35. Samples remained in the fixative at 4 °C for at least 24 h before further processing. They were then washed three times for 5 min each with 0.15 M HEPES, and postfixed with 1% OsO4 (EMS) in 0.1 M Na-cacodylate-buffer (Merck) at 4 °C for 1 h. Thereafter, the cells were washed three times for 5 min each in 0.1 M Na-cacodylate-buffer and dehydrated in 70%, 80%, and 96% ethanol (Alcosuisse) for 15 min each at RT. Subsequently, cells were immersed in 100% ethanol (Merck) three times for 10 min each, and then submerged in acetone-Epon (1:1) overnight at RT. The next day, the cells were embedded in Epon (Fluka) and left to harden at 60 °C for 5 days. Sections were generated on an ultramicrotome UC6 (Leica Microsystems, Vienna, Austria): first semi-thin sections (1 μm) for light microscopy, which were stained with 0.5% toluidine blue O (Merck), and then ultrathin sections (70–80 nm) for electron microscopy. The sections were mounted on single-slot copper grids and stained with uranyl acetate and lead citrate using an ultrastainer (Leica Microsystems). Sections were analyzed with a transmission electron microscope (CM12, Philips) equipped with a digital camera (Morada, Soft Imaging System) and image analysis software (iTEM).

### Statistics

All data are presented as means ± standard deviation (SD). Two-tailed unpaired Student’s t-test was used to assess significance of differences. Statistical significance was defined as follows: *P < 0.05, **P < 0.01, ***P < 0.001. The number of repeats performed for each experiment varied between three and eleven. The exact number is indicated in the corresponding figure legend.

## Results and Discussion

### Lung-on-chip with a passive exchange mechanism

A 3.5-μm porous PDMS membrane was integrated in this lung-on-chip device. To our knowledge, this is the thinnest porous PDMS membrane used in an organ-on-chip device to date^6,8,30,31,43^. The thickness of 3.5-μm was chosen as the best compromise between structural integrity and thickness, and can be fabricated reliably and reproducibly. PDMS was used due to its excellent elastic properties^44^ and biocompatibility^45,46^. The membrane covers a circular area with a diameter of 3 mm. The low area decreases the number of primary cells necessary to establish a confluent barrier, while at the same time providing sufficient material for downstream processes such as PCR. Due to its thinness and relatively large surface area, the membrane is highly sensitive to pressure gradients. Therefore, the biggest challenge of the new lung-on-chip design was to integrate a perfusion concept that is simple in application, but does not irregularly deflect the thin porous membrane during medium exchange.

**Figure 1:**
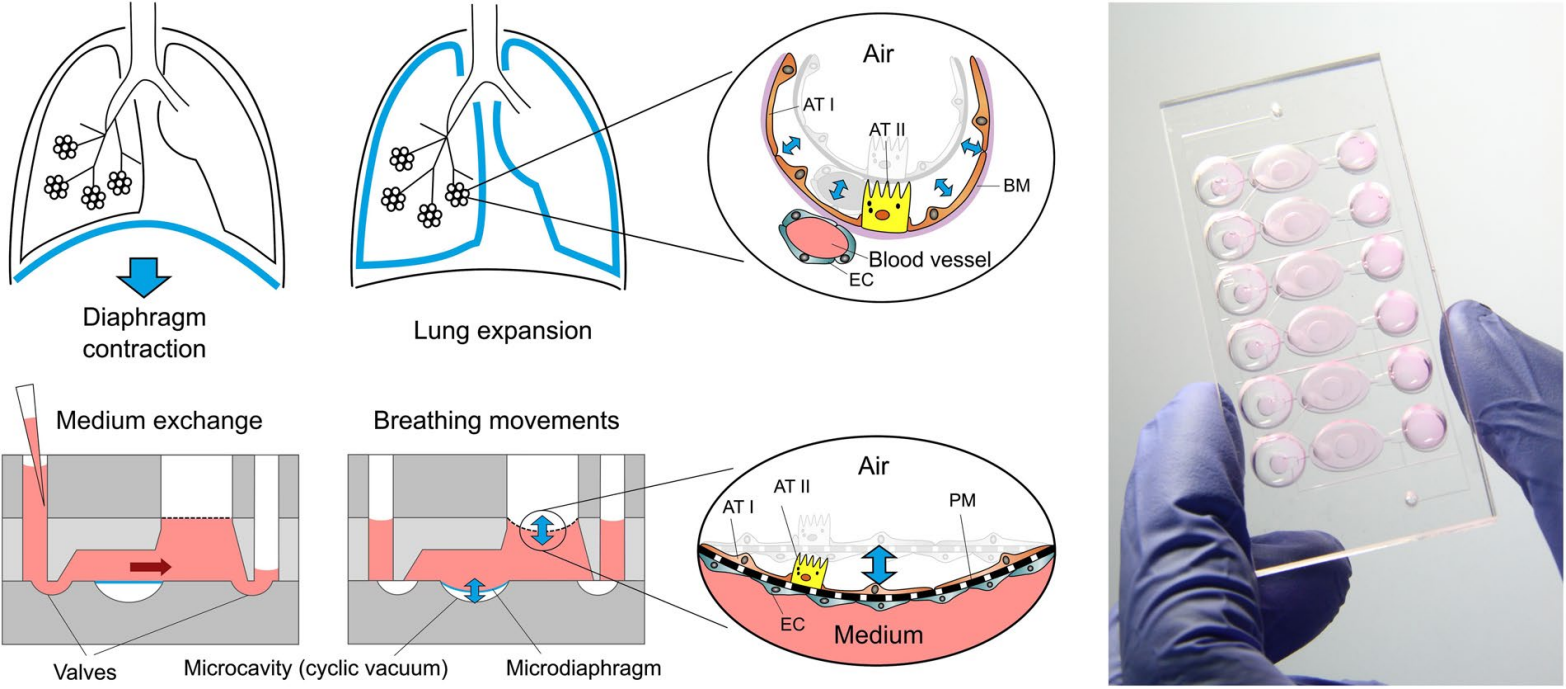
Concept of the newly developed lung-on-chip. Top: *in-vivo*, the lung expands following the contraction of the diaphragm. The breathing motions are transferred from the organ-level to the individual alveoli. The alveolar barrier consists of a tight alveolar epithelial cell layer – made of type I (AT I) and of type II (AT II) alveolar epithelial cells – and of endothelial cells (EC) between which the basal membrane (BM) is sandwiched. Bottom: Schematic cross-sections of the lung-on-chip with two operation modes: (i) Breathing and (ii) Medium exchange modes. The breathing motions of the alveolar barrier are induced by a bio-inspired microdiaphragm. When a cyclic vacuum is applied in the microcavity, the microdiaphragm is deflected. Two valves located on each side of the basal compartment can be opened to exchange the cell culture medium. Hydrostatic and surface tension forces transport the flow. After the exchange the valves are closed and the supernatant can be sampled from the outlet. Right: Photograph of the lung-on-chip with 6 independent alveolar barrier systems filed with cell culture medium.

To monitor the deflection of the thin membrane during medium exchange, the forces acting on the membrane need to be precisely controlled. Although this could be done with syringe or peristaltic pumps^6,47^, or the use of, e.g., tilting tables^14,48^, this would increase the overall handling complexity and error rate. From the standpoint of simplicity, we decided to integrate a passive exchange mechanism based on hydrostatic and surface tension forces. During medium exchange, these two forces counteract each other on the membrane, allowing us to control membrane deflection by defining the flow resistance of the microchannels before and after the membrane. These critical flow resistance values could be appropriately defined using a mathematical model of medium exchange inside the chip (see SI: Mathematical modeling). The final design of the lung-on-chip array includes six wells, each of them representing an independent alveolar barrier system (Fig. 1). This design allows two to six times more experiments to be performed than on previously developed breathing lung-on-chip devices^6,30^. The chip is composed of a fluidic and pneumatic part, which can be assembled reversibly (Fig. S1). This two-part setup facilitates cell seeding on either side of the membrane, as described previously^30^.

Figure 1 shows a schematic cross-section of one alveolar barrier system including all features described above. A detailed description of the dimensions and mode of operations is given in the Supplementary Information. The fluidic part includes an inlet and outlet well, the porous membrane, a microchannel that connects the inlet and the outlet, an apical cell culture well, and a basal cell culture chamber; the well and chamber hold a volume of 80 μL each. The pneumatic part is composed of two valves and a micro-diaphragm. The chip has two different operation modes: (i) breathing and (ii) medium exchange. During breathing, the valves are closed, and the micro-diaphragm is cyclically deflected via an external electro-pneumatic setup. Because fluid is incompressible and the basal chamber is closed, the movements of the micro-diaphragm are directly transferred to the thin membrane. In this way, it is possible to recreate the three-dimensional breathing motions. In medium exchange mode, breathing is stopped, and the inlet well filled with fresh medium. The valves are then opened by applying negative pressure via the external electro-pneumatic setup. The medium in the basal chamber is exchanged passively by means of hydrostatic and surface tension forces alone. After the medium is exchanged, the valves are closed again. The used medium (basal supernatant) can be collected from the outlet well and used for further analysis. In the final step, the inlet and outlet wells are emptied, and breathing mode is restarted.

This new lung-on-chip device, with a passive exchange mechanism, is simple to handle and enables long-term breathing of co-cultures at air–liquid interface, up to 22 days. Furthermore, because no external tubing or pumps are used for the perfusion, the risks of contamination, leakage, and air bubbles are nearly eliminated^46,49,50^. Additionally, the semi-open design makes it easy to create and maintain air–liquid cell culture conditions, which is usually difficult inside small microfluidic channels in combination with elastic membranes due to high surface tension forces^51^. Furthermore, it ensures similar gas supply as for conventional cell-culture dishes placed in the incubator.

### Lung-on-chip design validation

After mathematical modeling, chip design and fabrication, the lung-on-chip was experimentally tested for its functionality. In the first step, the chip had to be assembled and initially filled. To close the chip, the pneumatic part was aligned with the fluidic part and reversibly closed by manual pressure. The reversible closing of the chip allows separation of the different parts at the end of the experiment, enabling high-resolution imaging. A working lung-on-chip requires successful chip sealing and initial filling. Fortunately, both can be tested in one step, because if the chip were not sealed after closing, it would not be possible to fill it. To evaluate this issue, the chip was reversibly closed, and basal chamber filling was recorded under the microscope. The specific 3D design of the basal chamber, as well as the initial hydrophobicity of the PDMS, enabled filling of the chip without incorporation any air bubbles (Fig. S2). Successful filling proved that the chip was well sealed. One might also consider testing the delamination pressure of the chip; however, because we were creating negative pressure inside the basal cell culture chamber, delamination could not occur during normal chip operation. The lung-on-chip was designed to generate about 8% linear strain, well within the physiologically relevant range of 5–12% strain *in vivo*^52,53^. The measured linear strain inside the lung-on-chip was 7.6 ± 0.66% on average (see Fig. S3).

**Figure 2:**
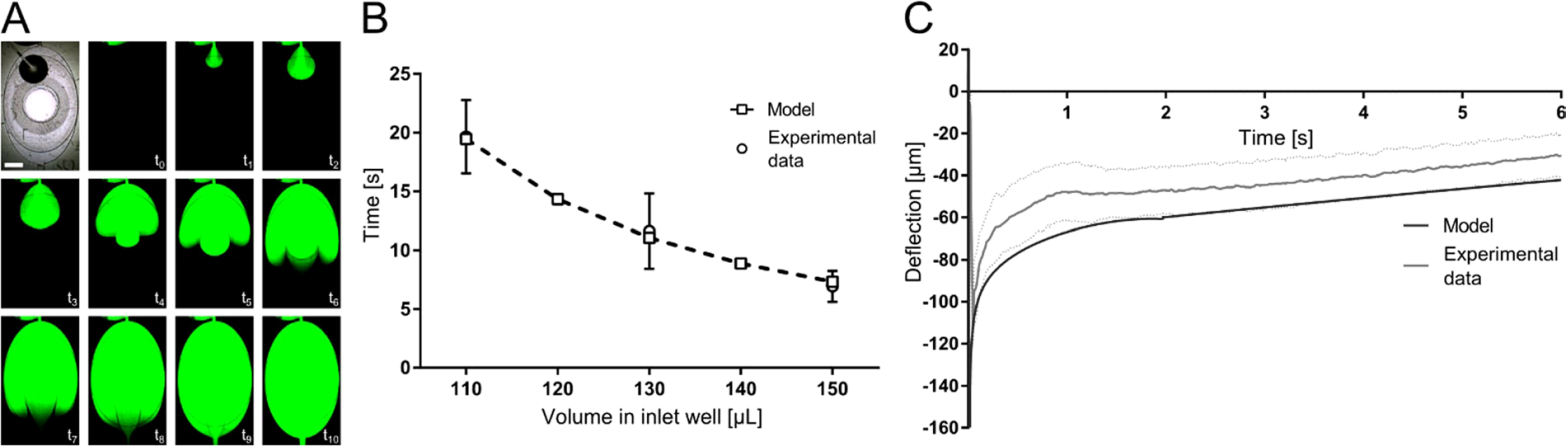
Characterization of the lung-on-chip array. (**A**) Time sequences of the medium exchange, visualised with a fluorescence dye (FITC-Sodium in PBS). Scale bar: 2 mm. (**B**) Comparison of the experimental and theoretical medium-exchange time as function of the volume pipetted in the inlet well (n = 4–7). (**C**) Comparison of the deflection of the thin porous membrane during medium exchange between experimental data and the mathematical model data (n = 11).

In the next step, medium exchange was tested and quantified. The medium in the apical well can be simply exchanged using standard pipettes. In the basal chamber, the medium is exchanged via the passive mechanism. Figure 2A depicts replacement of the cell culture medium in the basal chamber with fluorescein solution. The image sequence shows that the medium was exchanged efficiently without introducing any air bubbles.

We then tested the lung-on-chip to determine whether it works as mathematically modeled. To this end, we analyzed the dependency between hydrostatic pressure and the time needed to exchange the medium was analyzed. To vary the hydrostatic pressure, different volumes of cell culture medium were pipetted into the inlet well. Then, the valves were opened, and the exchange times were measured; these experimental times were then compared to the predictions of the mathematical model. The results of this comparison are shown in Fig. 2B. The experimental times corresponded very well with the predictions, indicating that the chip worked as proposed.

To further confirm this, we quantified the movement of the thin membrane during medium exchange. To measure the fast deflection of the thin porous membrane during the exchange, we developed an optical measurement technique (Fig. S4). Using this technique, the deflection as a function of time could be measured experimentally. Figure 2C shows a comparison between the predicted membrane deflection and the experimental data. The results show that the mathematical model slightly over-estimates the membrane deflection, possibly due to differences in the Young’s modulus of PDMS or small variations in the dimensions of the membranes tested. Nevertheless, the experimental deflection pattern corresponded well to the prediction of the model. Thus, these results provide further confirmation that the lung-on-chip is working as intended. The maximal deflection of the thin, porous membrane during exchange was −112 μm, corresponds to less than 0.4% linear strain. This small strain can be considered negligible if the cells are stretched to simulate breathing motions, during which the cells are exposed to ~8% linear strain. By contrast, if the chips are kept under static conditions, this weak strain might lead to a short-term cell response. However, because the cells are cultured over multiple days, and the medium is exchanged only daily or every second day, this effect could easily diminish over time. In addition, once endothelial and/or epithelial cells are cultured on the membrane, the deflection will be even smaller due to the increase in overall stiffness. Therefore, we can neglect the possible effect of strain resulting from medium exchange on the cellular response. The mathematical model was also used to calculate the shear stress acting on the cells during exchange (Fig. S5), which was determined to be only 5 × 10^−1^ dyne cm^−2^. This is one order of magnitude smaller than the shear stress observed in human microvessels (3–10 dyne cm^−2^)^54^, and is thus unlikely to affect the cells.

### Validation of cell culture on chip

**Figure 3:**
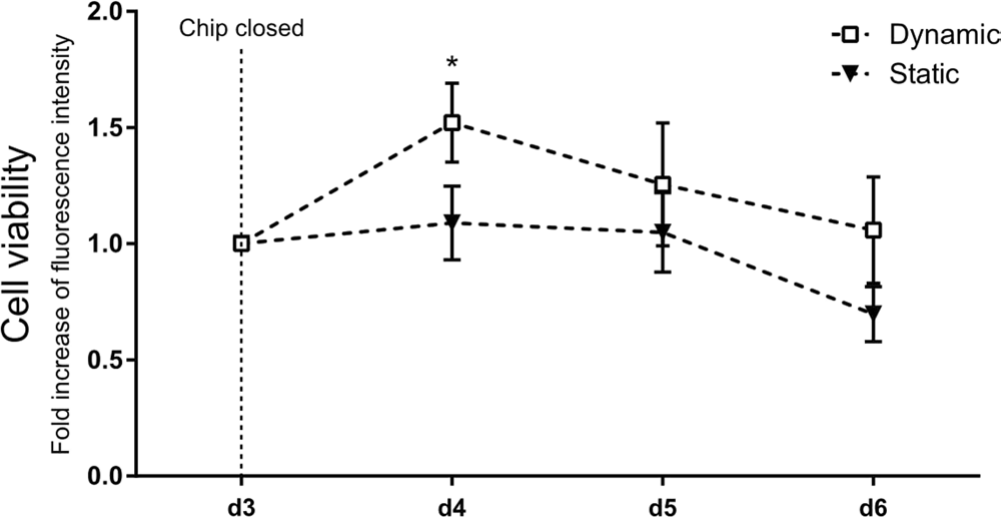
Cell culture validation on the breathing chip. Experimental handling of the new lung-on-chip array was tested with a commercially available viability test and the 16HBE14o- cell line. Breathing mode (dynamic) was started on day 3. 16HBE14o- cells demonstrate under stretch a slightly increased viability (n = 3).

In an initial step, we tested the utility of the new device and compared daily chip handling routines, such as cell seeding, medium exchange, and stretching. 16HBE14o- cells were cultured on the chip for a total of 6 days. Medium was exchanged every day as outlined above. After 3 days, the chip was closed, and the cells were cultured under either static or dynamic conditions. We analyzed cell viability using the PrestoBlue® assay. For the readout, the apical supernatant was transferred to a separate 96-well plate. As shown in Fig. 3, 24 h of cyclic physiological stretching was sufficient to induce a significant increase in cell viability in comparison to static conditions. Viability continued to improve until the experiment was stopped on day 6. As expected, the integrated passive medium exchange mechanism simplified the establishment and maintenance of long-term cell cultures, and the results were comparable with previously reported data^55^. The readout could also be performed by placing the chip directly into the TECAN reader (Fig. S6).

### Primary human alveolar epithelial cells on chip

A major challenge for microfluidic devices is the cultivation of primary cells^46,56^, which are often very stress-sensitive and unable to proliferate *in vitro*. Consequently, to generate a confluent monolayer, seeding densities and coatings must be optimized. For establishment of an advanced alveolus-on-chip, it is necessary to use primary alveolar epithelial cells (ATI and ATII cells), which play distinct roles in the alveolar microenvironment. ATI cells are large, flat, intricately branched cells with multiple cytoplasmic leaflets, and are almost devoid of organelles. Their main function is to provide an extensive, thin alveolar barrier that enables optimized (gas) transport^21,57^. On the other hand, the main functions of ATII cells are the synthesis, storage, and release of pulmonary surfactant into the alveolar hypophase, where it improves lung compliance and prevents alveolar collapse^58^. ATII cells also regulate the alveolar compartment and respond to alveolar injury by proliferating, serving as progenitors for both type I and type II cells^59^. The two cell types play different roles in alveolar fluid and ion transport^60–62^, reflected by their distinct expression patterns of various aquaporins and tight junctions proteins^63,64^.

**Figure 4:**
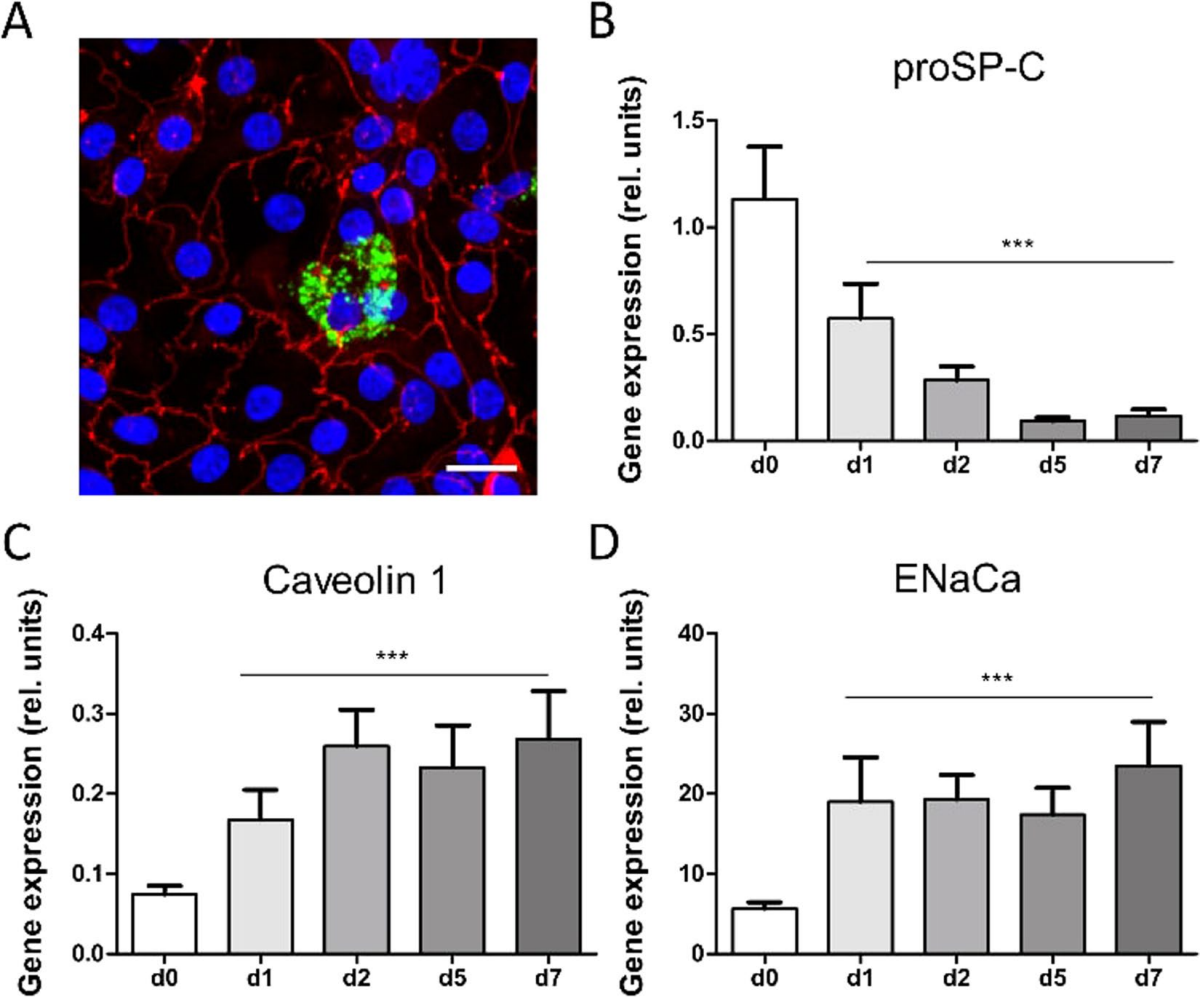
Cell differentiation on the chip. (**A**) After 5 days in culture, primary alveolar epithelial cells were stained against surfactant-protein (SP)-C, (marker of alveolar type (AT)-II cells, green) and Zonula occludens (Zo)-1 (marker for tight junctions, red). ATII-like cells specifically expressed SP-C (green). ATI-like cells were characterized with flat and enlarged cell bodies. Scale bar: 20 μm. (**B**–**D**) Gene expression analysis of primary human alveolar epithelial cells (hAEpC) on chip over 7 days indicated a decrease of ATII-cell marker (proSP-C), an increase of ATI-cell marker (caveolin-1) and the epithelial sodium transport channel (ENaCα). Gene expressions for all days on chip were compared to freshly isolated cells at D0 (n = 6, each time point).

In this study, we achieved successful cultivation of hAEpCs on a chip. Upon seeding, we observed good attachment of freshly isolated ATII cells (day 0)^40,65^. After 2 days on the chip, the cell monolayer reached confluence, and on day 5 the majority of cells possessed an ATI-like phenotype with high expression of ATI cell marker caveolin-1 and the tight junction protein Zo-1 (Fig. 4A, red staining). In addition, a small fraction of ATII-like cells was detected by immunofluorescence staining against mature SP-C (Fig. 4A, green label) or the ATP-binding cassette transporter (ABCa3). Both proteins are selective markers for ATII cells and specifically localized in lamellar bodies (LBs), which are storage vesicles for pulmonary surfactant^57,66,67^. Furthermore, gene expression analysis of cells on chips under static conditions revealed that expression of proSP-C decreased, whereas gene expression of the ATI-specific protein caveolin-1^65,68^ increased, over time (Fig. 4B,C). These observations on the gene expression level were confirmed by counting the total number of cells on the well and analyzing the ratio of mature ABCa3 positive stained cells on day 1 and day 5 (Fig. S7). Expression of the apical sodium transporter EnaCα, designating polarized cells with active water transport capacity (Fig. 4D), also increased. Similar results were reported in previous studies, suggesting that ATI cells *in vivo* play an important role in lung fluid homeostasis^61,68^. The fine equilibration of the alveolar lining fluid is decisive for lung function, and fluid maladjustment impedes gas transport and induces alveolar collapse due to high surface tension.

**Figure 5:**
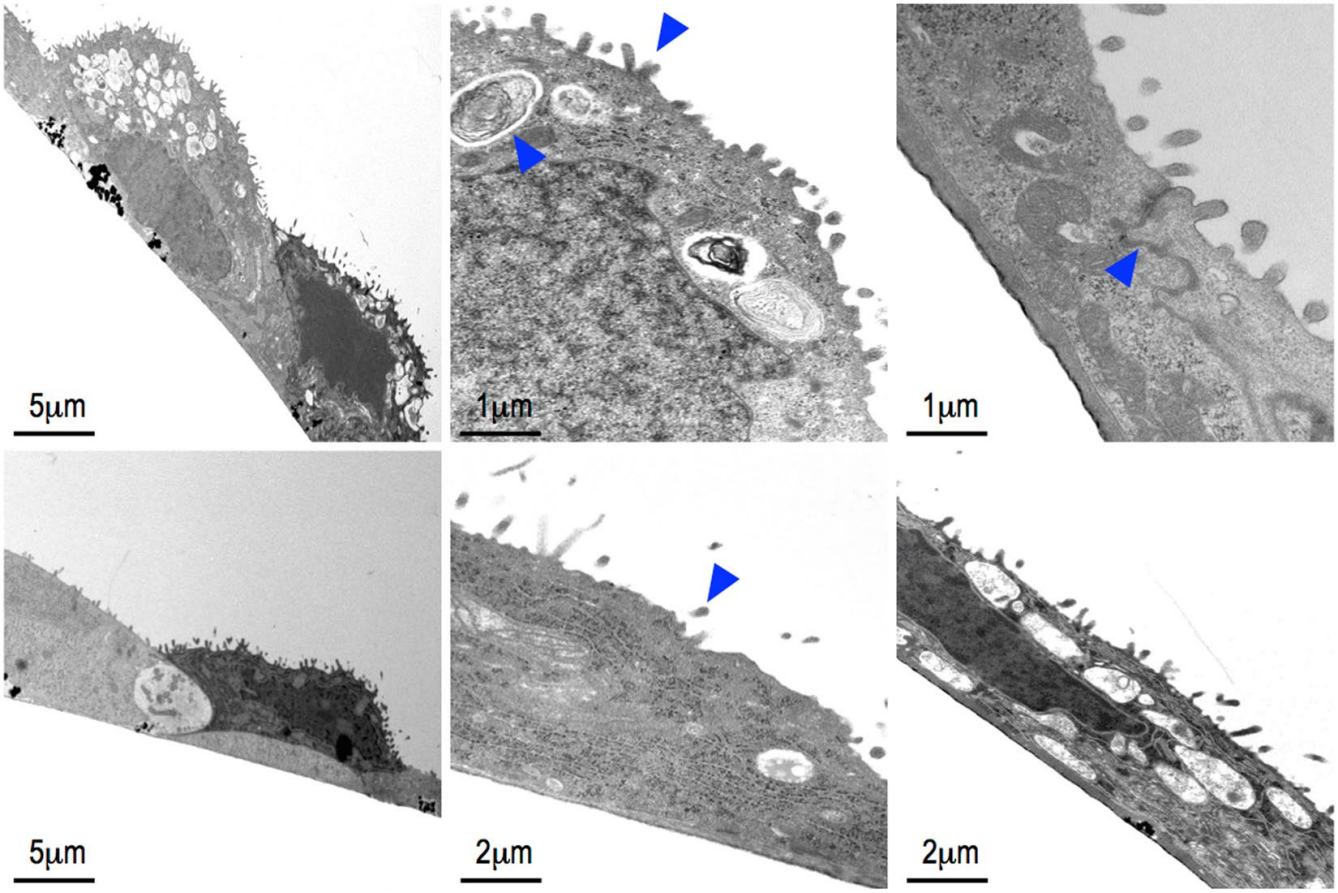
Transmission electron micrograph of air-liquid interface exposed human alveolar epithelial cells (hAEpC) in in static conditions (**A** i-iii) and dynamic conditions (**B** i-ii). Major parts of the surface were covered with an ultra-thin film of ATI-like cell protrusions. ATII-like cells were filled with lamellar-body like vesicle (**A** ii, arrow). Microvilli structures (arrow **A** ii and **B** ii) and tight junctions (arrow **A** iii) indicate integrity of the cell layer.

Recent observations demonstrated that besides tissue stretch, the air compartment (and thus the associated surface tension) is the most important physiological stimulus for surfactant release^37,38,69^. Thus, the creation of a confluent epithelial monolayer at the air–liquid interface is a key prerequisite for tissue-specific cell differentiation on the chip. As shown in Fig. 4A, a confluent monolayer of hAEpCs formed at the air–liquid interface after 5 days. Micrograph sections (60–80 nm thick) revealed that most of the area was covered by flat, simply structured cells (see Fig. S8A) with large ultrathin cell protrusions (<2 µm, Fig. 5Aiii,Biii), as described previously by Weibel^21^ and Fuchs *et al*.^65^. The surface of ATI cells contains characteristic caveolae-like structures^65,70^, which we identified by immunofluorescence staining for caveolin-1 (Figs S7A and S8B). We also identified a round, much smaller cell type dispersed within the monolayer. These round cells contained a number of relatively electron-dense multi-lamellar vesicles (Fig. 5Ai,Bi, arrow). These LB-like vesicles generally appeared empty, potentially due to a methodological artefact arising during sample fixation. However, occasionally we were able to reconstruct specific phospholipid lamellae structures, indicating a functional and intact mature LB (Fig. 5Aii arrow)^71^.

The apical cell surfaces of both cell types were densely covered with tiny (200–500 nm) microvilli protrusions and high magnification revealed strong tight junctions between neighboring cells (Fig. 5Aii,Bii; arrow). Morphology of hAEpCs on chips were comparable to those reported by previous *in vivo* and *in vitro* studies^65,72^. Fig. 5Bi–iii show hAEpCs exposed to 72 h of stretching, from day 2 to day 5. Electron microscopic analysis revealed no apparent differences in cell differentiation and morphology between static and dynamic conditions. However, we must take into account the fact that these sample sections were only minute extracts of the total surface area of the chip, and the analysis was performed in a descriptive manner, focusing on general monolayer integrity and cell morphology. Future studies are required to achieve quantitative evaluation of how stretching affects proliferation and trans-differentiation of freshly cultured hAEpCs on chips. Furthermore, to optimize the ratio between ATI and ATII cells, it would be useful to systematically examine the cell culture protocol and conditions, including growth factor supplement, air–liquid interface treatment and stretch protocol, with the aim to reaching proportions comparable to those recently described by Weibel^21^. To our knowledge, this is the first time that ATI- and ATII-like cells have been co-cultured and identified on-chip, resembling an almost *in vivo*–like alveolar epithelium. The two cell types could clearly be distinguished from each other using selective markers and electron microscopy. These microscopic observations were strengthened with results obtained from gene expression analysis.

**Figure 6:**
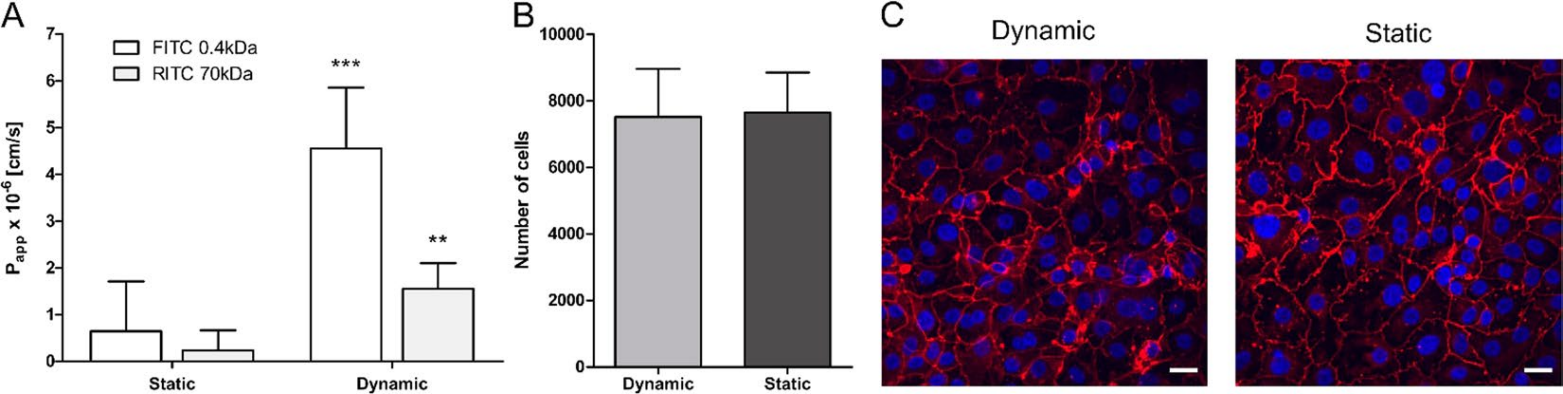
The effect of cyclic strain on the permeability of primary alveolar epithelial cells. (**A**) Apparent permeability for FITC and RITC for static and dynamic conditions. The relative transport of a small hydrophilic molecule FITC-sodium (4 kDa) and RITC-dextran (70 kDa) across the monolayer significantly increased upon a physiological cyclic strain. Cyclic stretch was applied for 72 hours and a control was kept under static conditions (n = 5–6). (**B**) Cell number of primary alveolar epithelial cells in dynamic and static conditions. The mean cell number was equal in dynamic and static conditions. We used a Fiji macro (Unsharp Mask, Threshold, make binary, watershed & analyse particles) to quantify the images obtained by scanning a whole chip (n = 4–6). The analysis revealed that the number of cells is not influenced by the physiological stretch. (**C**) Tight junction expression. The fluorescence images show the cell nuclei in blue and the tight junction protein Zo-1 in red. In both conditions, Zo-1 was well expressed, which confirmed a tight cell monolayer and that the physiological stretch of 8% linear did not negatively influence the cell monolayer integrity. The images were taken in the centre of the cell culture well, where the stretch effect is maximal. Scale bars: 20 μm.

Previously, we reported that mechanical strain increases the transport of small hydrophilic molecules (FITC 0.4KDa) in the 16HBE14o- cell line^30^. However, to reconstitute alveolar transport, implementation of a primary cell model is crucial, because most available alveolar epithelial cell lines (e.g., A549, R3/1, MLE-12, etc.) lack the ability to form tight junctions, resulting in low epithelial resistance and insufficient barrier function^73^. Therefore, we assessed the effect of cyclic physiological strain on the permeability properties of primary alveolar epithelial cells (hAEpCs) (Fig. 6A). These experiments revealed that the permeabilities of a small hydrophilic tracer, FITC-sodium, and of a macromolecule mimetic, RITC-dextran, were significantly increased when cells were exposed to 72 h of stretching from day 2 to day 5. Flux of FITC–0.4 kDa was about 7-fold higher under dynamic conditions (4.5 ± 1.3*10^−6^ cm/s) than under static conditions (0.65 ± 1.1*10^−6^ cm/s). The transport rate of RITC–70 kDa was increased to a similar extent, from 0.24 ± 0.43*10^−6^ cm/s (static) to 1.6 ± 0.54*10^−6^ cm/s (dynamic mode). To rule out that increased permeability is based on cell detachment and reduced cell number we performed whole-chip imaging and counted the total number of cells on the chip. As shown in Fig. 6B, the total number of cells was similar under static (7655 ± 1204) and dynamic (7516 ± 1444) conditions. Furthermore, cell monolayers on stretched membranes exhibited tight junction expression similar to that of non-stretched membranes (Fig. 6C). These data differ considerably from the results of our previous study: 16HBE14o- exhibited a much smaller (1.5-fold) increase in FITC–0.4 kDa permeability and no significant increase in RITC–70 kDa flux. This important difference in stretch sensitivity might be explained by differences in stretching time (21 h vs. 72 h) or the cell culture protocols used (submerged vs. air–liquid conditions), or the fact that 16HBE14o- are bronchial epithelial cells. It is also possible that primary alveolar cells are much more susceptible to mechanosensitive stimulation than cell lines^53^.

This is the first on-chip study evaluating the effect of enduring, cyclic physiological stretch (8%) on hAEpCs. Future studies should investigate the underlying signal mechanisms, which remain unclear. Notably in this regard, however, studies with primary rat alveolar cells demonstrated that 1 h of biaxial stretch of 37% increased the permeability capacity of small uncharged molecules and affected peripheral Zo-1 expression^74–76^. These *in vitro* results were verified in whole-lung experiments using FITC-Albumin (55 kDa) as a tracer. Interestingly, only 30 min of 12% stretch was sufficient to show a trend in permeability increase and with 37% biaxial stretch the effect was significant^76^. The authors reported that even low stretch magnitudes, in the physiological range, could induce similar cell responses if the exposure times were prolonged. Furthermore, the study revealed that stretching induces actin cytoskeleton reorganization, probably mediated by intracellular Ca^2+^ increase. This leads to multiplication of large cellular membrane pores, thereby increasing the transport of larger molecules like albumin^76^. These results emphasize the importance of assessing permeability under physiological breathing conditions.

**Figure 7:**
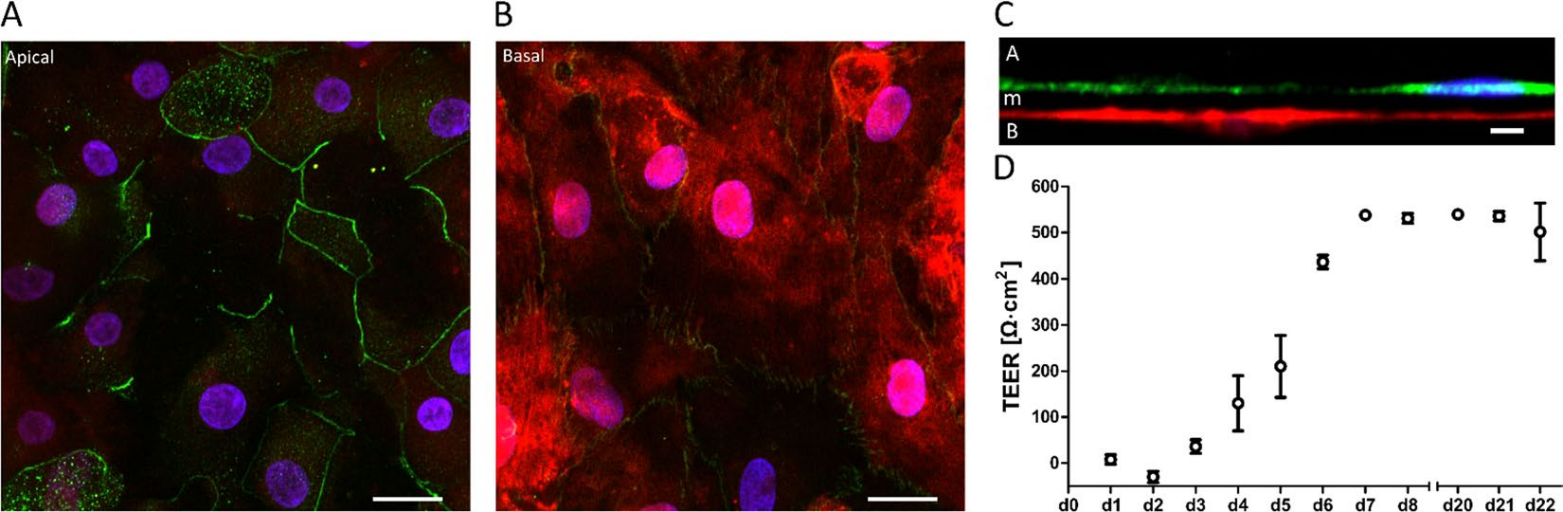
Long-term co-culture on chip. (**A**,**B**) Fluorescence micrographs showing the cell nuclei (blue) and tight junction protein Zo-1 (green) of primary human alveolar epithelial and primary human lung microvascular endothelial cells (fluorescently labelled in red) after 22 days in co-culture (**C**) Orthogonal view of the co-culture showing the epithelial cells on the apical side (**A**) in green (E-cadherin), the endothelial cells on the basal side (**B**) in red and the ultra-thin membrane in-between (m). (**D**) Long-term TEER measurements of the co-culture over 22 days show the maturation of the barrier integrity over time (n = 3). Scale bars: 20 μm, 20 μm, 10 μm.

As a next step towards an advanced alveolus-on-chip for drug transport studies, we recreated the alveolar air–blood barrier by establishing a co-culture of primary epithelial and endothelial cells. Previous studies showed that co-culture of epithelial and endothelial cells improves barrier properties and mimics *in vivo* signaling pathways in a more realistic way^72,77^. We managed to co-culture primary human alveolar epithelial (hAEpCs) and primary human lung microvascular endothelial cells (VeraVecs) over a 22-day time course. After 22 days in culture, hAEpCs and VeraVecs still expressed tight junction protein Zo-1 (Fig. 7A,B). The integrated 3.5-µm porous PDMS membrane allowed recreation of the very thin air–blood barrier *in vitro*, shown in Fig. 7C. The total local thickness of our barrier was less than 10 μm; for comparison, commercially available cell inserts routinely used for drug transport studies have a membrane thickness of 10 μm^78^ without cells. The thinness of our barrier brings us close to the *in vivo* dimension of the air–blood barrier, described by Gehr *et al*., with an alveolar arithmetic mean thickness of 2.2 µm^79^.

TEER measurements further revealed that barrier integrity matured over time, reaching a plateau after 7 days in co-culture (Fig. 7D); the maximal TEER value of 545.5 ± 2.86 Ω cm^2^ was reached at day 14. Although TEER in co-culture on our chip was smaller than previously described for hAEpCs in inserts (1000–2000 Ω cm^2^)^80^, these values reflected a tight monolayer enabling active water and selective ion transport^73,77^. Differences between TEER values measured in inserts and in microfluidic systems have been reported earlier and are thought to be due to a geometrical origin rather than a biological one^81^. Furthermore, TEER experiments often exhibit significant discrepancies, due to different culture conditions or (most probably) donor-to-donor variability^73,77^. Successful implementation of a non-invasive TEER measurement using standard equipment would enable live cell monitoring of cell growth, differentiation and barrier integrity, eliminating the need to integrate electrodes directly inside the chip, as previously reported^82–85^. After 22 days, when the culture was aborted, the co-culture and the barrier functionality were still intact. The experiments could have therefore been performed for longer periods of time for defined drug discovery or toxicity assay, such as acute inhalation toxicity studies^86^. To the best of our knowledge, this is the first study in which a co-culture of primary human lung epithelial and endothelial cells was established on a lung-on-chip device in a manner that enabled elevated TEER values to be established, maintained, and measured over a long period of time^6,80^.

## Conclusion

The breathing lung-on-chip array described here mimics several key aspects of the alveolar microenvironment, such as the breathing motion, the air-blood barrier and the air-liquid interface. It enables to culture and maintain primary human ATI and ATII-like epithelial cells from patients’ for several days. Long-term co-cultures including human alveolar epithelial cells and endothelial cells are also possible thanks to the integrated passive cell culture medium exchange. In addition, the simple handling of the device and its compatibility with several standard laboratory instruments are serious advantages in view to its adoption in research as well as in the pharmaceutical industry. This also enables to establish reproducible cell culture conditions and assay protocols on the chip. The present study demonstrates that this alveolus-on-chip array is a valuable tool to model the alveolar barrier. It has the potential to become an important tool in drug discovery and in personalized and precision medicine applications to optimize the therapy of each patient based on results obtained on-chip with the patients’ own cells.

## Electronic supplementary material

Supplementary Information
