# Supplementary material for: Medium throughput breathing human primary cell alveolus-on-chip model
Source: Sci Rep. 2018 Sep 25;8:14359. doi: 10.1038/s41598-018-32523-x (PMC6156575; doi:10.1038/s41598-018-32523-x)
Supplement: Supplementary file 1 — Supplementary Information [file 41598_2018_32523_MOESM1_ESM.docx]

Supplementary information

Medium throughput breathing human primary cell alveolus-on-chip model

Janick D. Stucki‡, Nina Hobi‡, Artur Galimov, Andreas O. Stucki, Nicole Schneider-Daum, Claus-Michael Lehr, Hanno Huwer, Manfred Frick, Manuela Funke-Chambour, Thomas Geiser, and Olivier T. Guenat

**Lung-on-Chip design and operation**

**Lung-on-Chip device**

The overall dimensions of the lung-on-chip device are 35 x 75 mm. The device hosts six lung-on-chips each equipped with an inlet, an outlet and a cell culture well, where a thin, porous and elastic membrane is located. To operate the lung-on-chip device two pneumatic access ports are included on each side of the chip. Each cell culture well is separated from the next by 9 mm that corresponds to the standard distance between rows of wells defined by the Society for Laboratory Automation and Screening (SLAS) (1). The lung-on-chip is made of two main parts, the fluidic and pneumatic part, as shown in figure S1 A, B.

(1) <https://www.slas.org/SLAS/assets/File/ANSI_SLAS_4-2004_WellPositions.pdf>

**Fluidic part**

The fluidic part of the lung-on-chip is made of a top plate and a microfluidic middle plate. They comprise six individual lung-on-chip, each with a cell culture well, an inlet and an outlet. Both plates also include two access ports, one for the valves and one for the microdiaphragm (breathing mode). For details see figure S1 A. The top plate and middle plate are 2 mm and 1.5 mm thick, respectively. A thin, porous and elastic PDMS membrane is located between the top and middle plates, separating the cell culture well from the cell culture chamber. The membrane is 3.5 µm thin and has 3 µm circular pores with a pore density of 800’000 pores/cm^2^. The diameter of the cell culture well, where the porous membrane is located, is 3 mm. The volume of the cell culture well (apical side) and that of the cell culture chamber (basal side) are each 80 µl.

**Pneumatic part**

The pneumatic part is made of a 2 mm thick structured base plate and a 40 µm thick PDMS membrane. The base plate includes 12 microcavities for the valves as well as six breathing cavities. The 12 microcavities are connected to microfluidic channels ending in an access port. The same is true for the six breathing cavities. For details see figure S1 A.

**Lung-on-Chip operation**

The chip is pneumatically connected via the access ports and tubings to an electro-pneumatic setup (see figure S1 C). The electro-pneumatic setup allows to open and close the valves, by applying negative and positive pressures through the access ports of the valves. The applied pressure deflects the 40 µm thick PDMS membrane within the respective cavities, which results in the opening and closing of the valves. The setup also regulates the breathing motion by applying a negative pressure to the breathing access ports as a function of time (the pressure function is a triangle waveform). Again, the applied vacuum induces a movement of the 40 µm thick PDMS membrane (the microdiaphragm), which results in the generation of the cyclic breathing motion. The movements of the microdiaphragm are transferred to the thin, porous alveolar membrane. As shown and explained in detail in figure S1 D, the lung-on-chip has two operation modes: breathing and medium exchange modes.”

Supplementary Figure 1. The lung-on-chip with its structures and functions. A: Exploded-view of the lung-on-chip showing all features of each plate. The numbers in brackets indicate how many of the specific features are included in the lung-on-chip. B: Schematic cross-section of one alveolar barrier system of the lung-on-chip, showing the important design elements. C: Photograph of the lung-on-chip connected to pneumatic tubings. The two tubings, one for the actuation of the valves and one for the actuation of the microdiaphragms, are connected to an electro-pneumatic system that controls the applied pressures. Scale bar: 5mm. D: Schematic drawings of the two operation modes: i) breathing and ii) medium exchange modes. In the breathing mode the valves are always closed. The alveolar membrane is cyclically deflected in response to the movements of the microdiaphragm that is controlled by the applied a negative pressure as a function of time. ii) In the medium exchange mode the breathing is stopped. Fresh medium is pipetted in the inlet wells (1). Afterwards the valves are opened (2) and the cell culture medium is exchanged passively (3). Once the levels of the cell culture medium stabilize in the inlet and outlet well, the valves are again closed and the supernatant in the outlet wells can be sampled for further analysis (4).


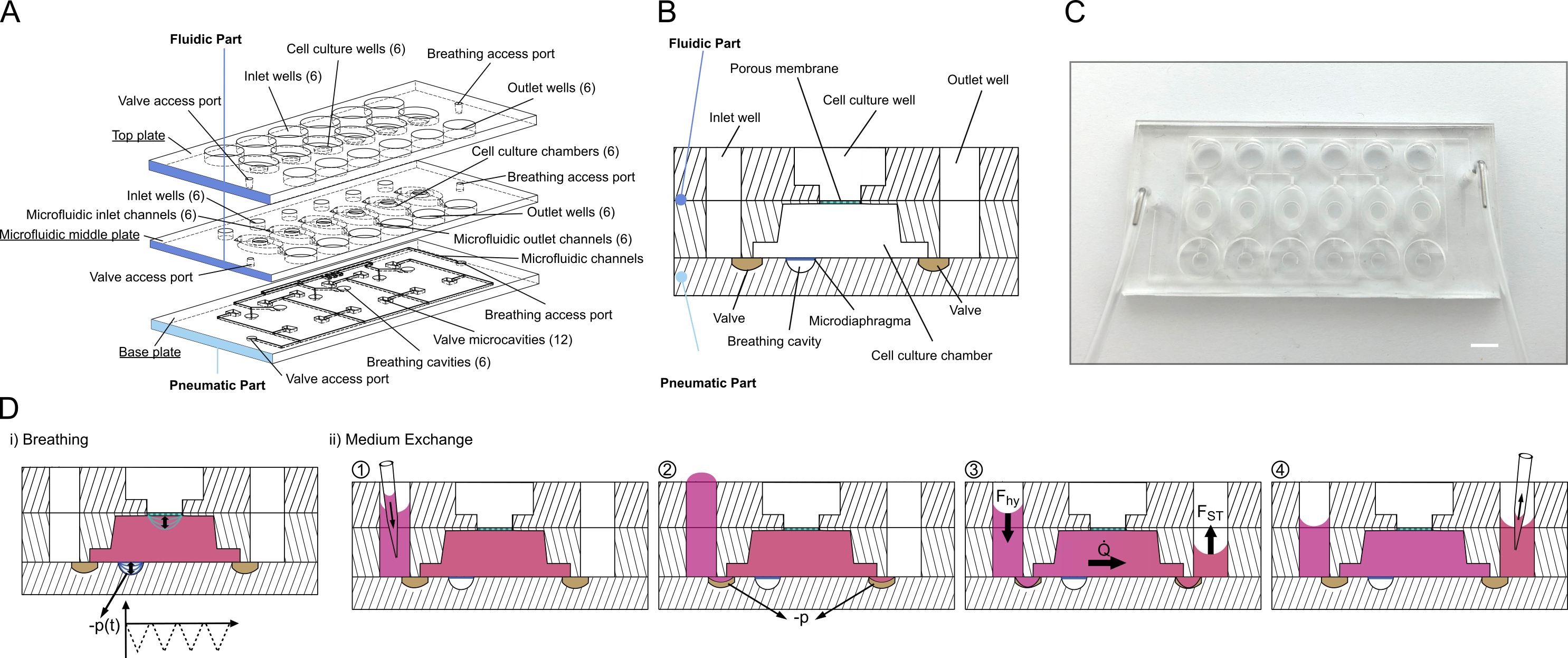


**Mathematical modelling**

The mathematical model was implemented in Matlab. To model the exchange and the resulting deflection of the thin membrane, we have to calculate the pressure (p_k_) in the cell culture chamber. This pressure (p_k_) can be derived from the equivalent electric circuit and the resulting equations:


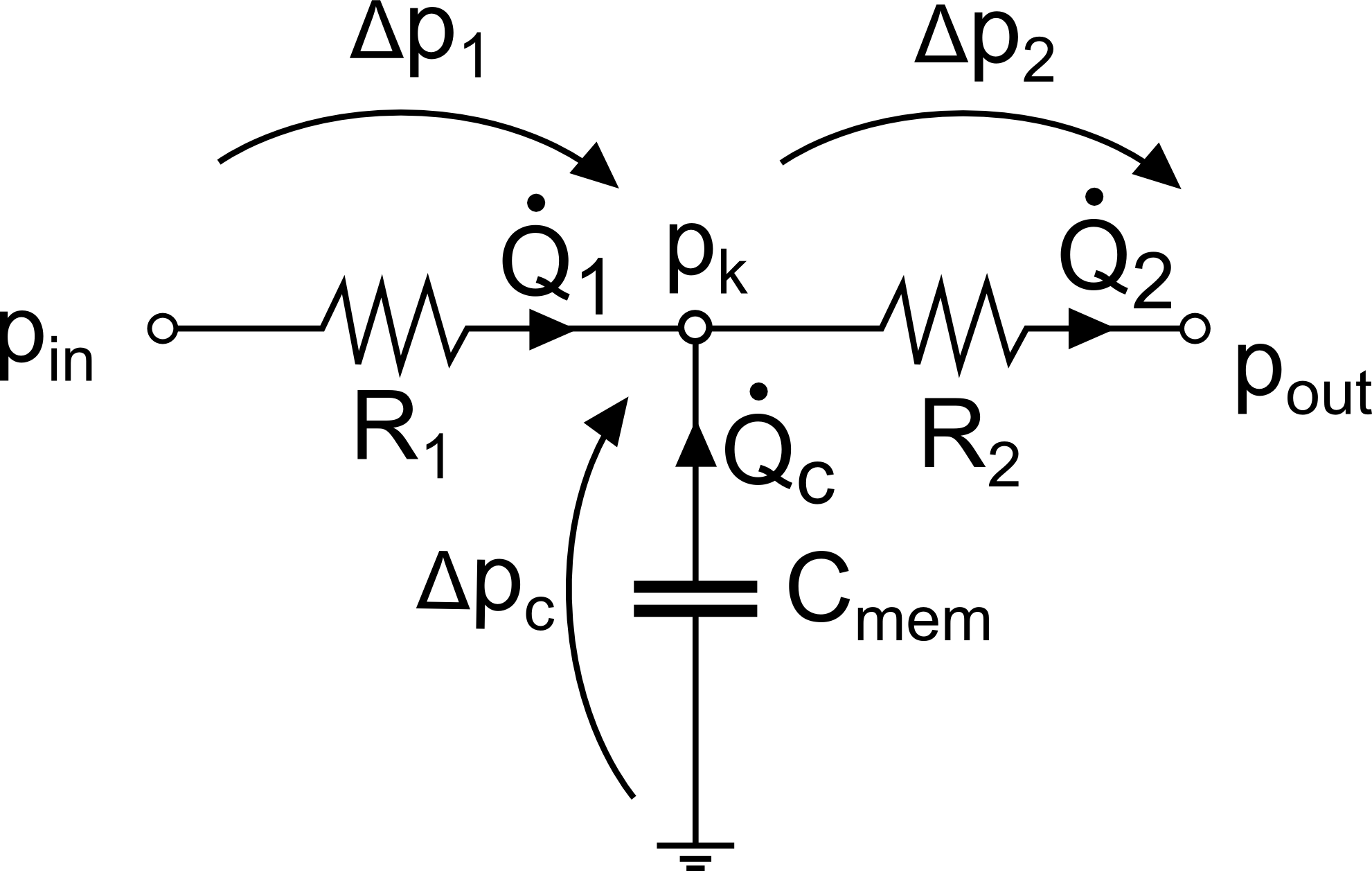


Equivalent electric circuit to model the medium exchange and the membrane deflection.

$${\Delta p}_{1}=p_{in}-p_{k}=\dot{Q}_{1}*R_{1}$$

$${\Delta p}_{2}=p_{k}-p_{out}=\dot{Q}_{2}*R_{2}$$

$\dot{Q}_{c}=C_{mem}*\frac{dp_{c}}{dt}=-C_{mem}*\frac{dp_{k}}{dt}$

$$\dot{Q}_{2}=\dot{Q}_{1}+\dot{Q}_{c}$$

🡺 $C_{mem}R_{2}*\frac{dp_{k}}{dt}+p_{k}\left( 1+\frac{R_{2}}{R_{1}} \right)-p_{out}-\frac{R_{2}p_{in}}{R_{1}}=0$

🡺 $p_{k}\left( t \right)=-\frac{D}{B}e^{-\frac{B}{A}t}+\frac{D}{B} with A=C_{mem}R_{2}, B=1+\frac{R_{2}}{R_{1}} \& D=p_{out}+\frac{R_{2}p_{in}}{R_{1}}$

To account for the time dependent changes in hydrostatic and Young-Laplace pressures in the inlet and outlet well, we defined first the initial p_in_=p_hy_ + p_YL_ according to the pipetted volumes (t=0) and p_out_=0. Then p_k_ is calculated as well as the resulting volumetric flowrates for one time step (1 ms). From the volumetric flowrates, the volume in inlet and outlet well were then calculated and the corresponding p_in_ and p_out_ adjusted.

$$p_{in}=p_{hy}\left( t \right)+p_{YL}(t)$$

$$p_{out}=p_{hy}\left( t \right)+p_{YL}(t)$$

This was then continuously done for each following time step.

The capacitance C_mem_ depends on the pressure p_k_, which was not considered in the differential equation above. To account for this dependency the capacitance was calculated at each time step from the p_k_ value of the previous time step. The capacitance was modelled as described previously by Inman et al. [1] (C_mem_=dV/dp). From experimental load deflection data of a 3.5μm thin PDMS membrane, we fitted the material properties (σ_0_, E) of the PDMS membrane using a Neo-Hookean material model[2]. This model defines the relationship between the pressure and the deflection of the circular PDMS membrane:

$$p = 4*\frac{\frac{w}{r}}{\left( r*\left( \left( \frac{w}{r} \right)^{2}+1 \right) \right)}*\left( \sigma_{0}*t_{m} +\frac{E*t_{m}}{3} * \left( 1-\frac{1}{\left( 1+\left( \frac{w}{r} \right)^{2} \right)^{3}} \right) \right)$$

where *w* is the deflection, *r* the radius and *t_m_* the thickness of the membrane.

The volume of the deflected membrane was modelled as spherical cap:

$$V = \pi*\frac{w}{6} * (3*r^{2}+w^{2})$$

Taking the derivative of the above two equations with respect to the deflection w, we can calculate the capacitance C_mem_ =dV/dp. The capacitance C_mem_ is as function of the deflection resp. the pressure (knowing the pressure-deflection relation). This relation was then used to define the corresponding C_mem_, depending on p_k_.

The viscosity of the fluid for the simulation of the exchange time (and all other simulations as well) was defined to be μ= 1*10^-3^ Pa s. Regarding the calculation of the exchange time, this assumption is reasonable since the chip was first filled with cell culture media + 10% FBS (μ=0.97*10^-3^ Pa s at 37°C [3]) and then exchanged with a FITC-PBS solution (μ≈ 1*10^-3^ Pa s at 20°C [4]). The surface tension was set to 5.26*10^-2^ N/m [5] and the contact angle to 10°, which represents a PDMS surface wetted/ coated with cell culture media including FBS (experimentally measured).


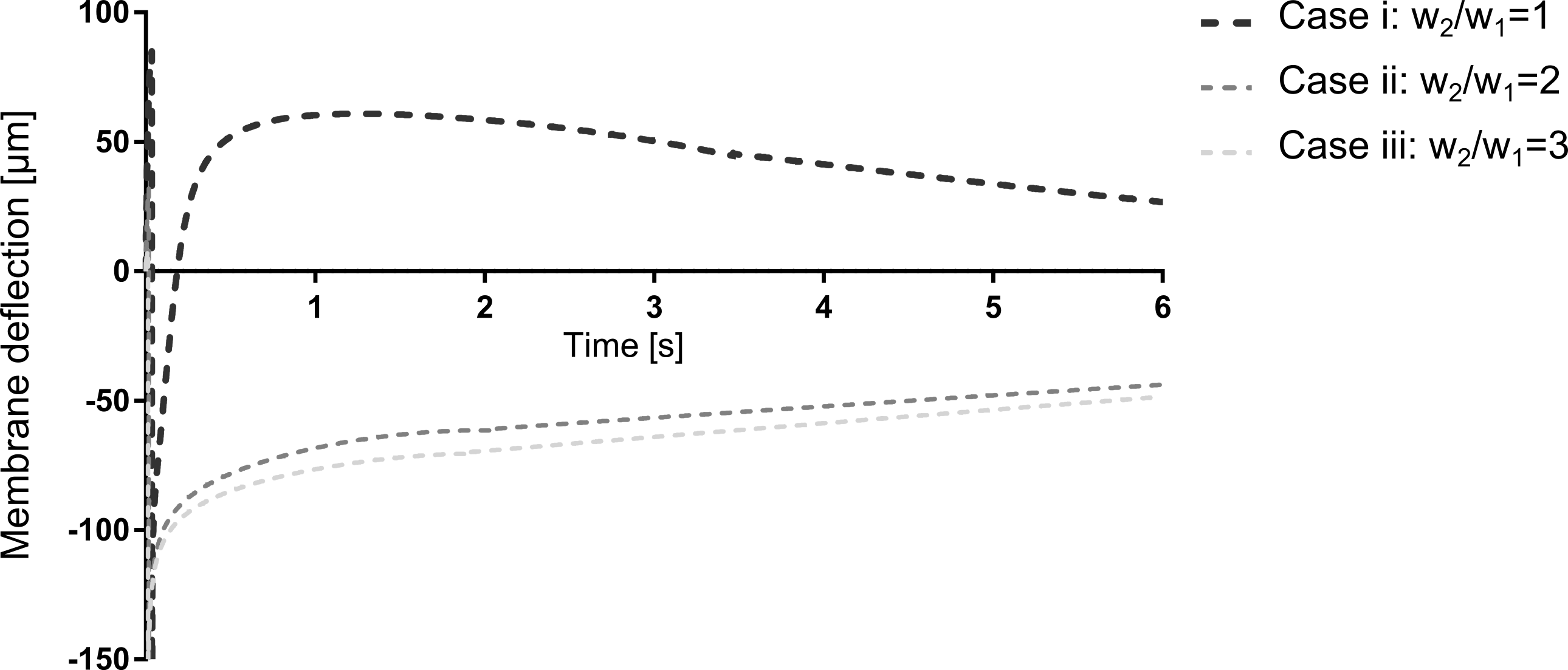
The model was used to evaluate the effect of different fluidic resistances of the inlet (R_1_) and outlet channels (R_2_) on the deflection of the thin membrane and on the exchange time. We fixed the width of the inlet channel (w_1_) and varied the width of the outlet channel (w_2_) to change the resistances. The deflection of the thin membrane as well as the exchange time was then calculated for three different flow resistance/ width ratios: i: w_2_/w_1_=1, ii: w_2_/w_1_=2 and iii: w_2_/w_1_=3. The results in the figure below show that the choice of fluidic resistances is crucial to control the deflection of the thin membrane. If the resistance of the outlet channel is too large (case i), the membrane is first deflected downwards (initial capillary forces) but then upwards (hydrostatic force) again. Afterwards, the deflection of the membrane gradually decreases. In the cases ii and iii, the capillary forces dominate the hydrostatic force. This means that the membrane is only deflected downwards and then gradually levels up, due to the hydrostatic pressure force. In these two cases the maximal deflection of the membrane is slightly larger, compared to case i (141.1 & 145.7 μm vs. 109.2 μm), but the membrane is only deflected in one direction. In both cases the induced mechanical strain would be as small as ε_ii_=0.59% and ε_iii_=0.63%, respectively. The time required to exchange the cell culture medium is in case I 12.87s, whereas in the cases ii and iii, it is 7.67s and 7.06s, respectively. Based on these results, we choose the ratio ii (w_2_/w_1_=2) for the chip design. It allows a fast exchange with a small deflection in only one direction.

**Initial chip filling**


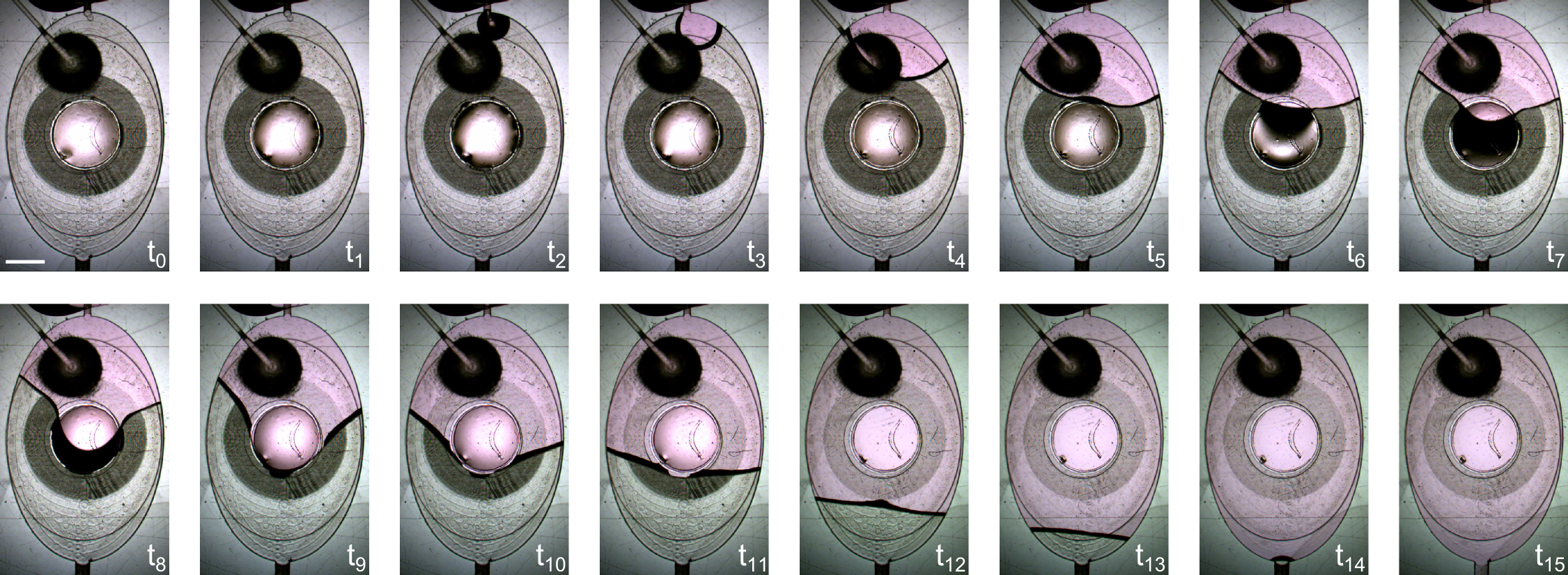


Supplementary Figure 2. Time sequences of the initial filling of the lung-on-a-chip array with cell culture media. After the pneumatic and fluidic part were assembled the lung-on-chip can be filled with cell culture medium. The design as well as the initial hydrophobicity of the PDMS allows to fill the chip without the incorporation of air bubbles. Scale bar: 2mm

**Strain inside the lung-on-chip**


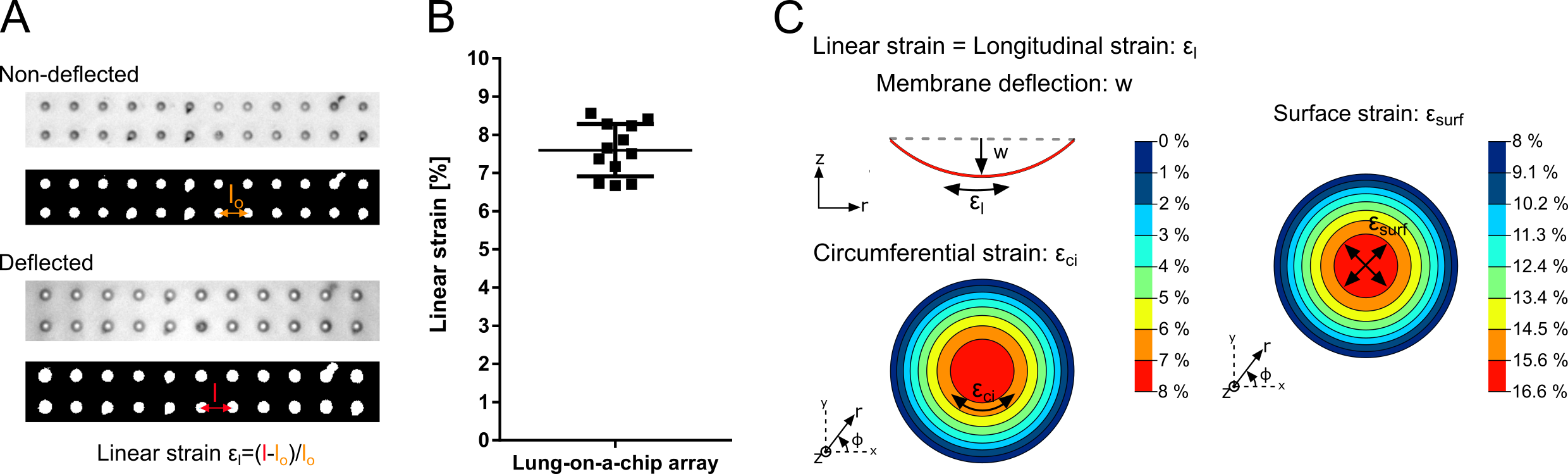


Supplementary Figure 3. Quantification of the applied linear strain in the lung-on-a-chip array and differences between strain and deflection. A: Shows representative micrographs and the corresponding thresholds of the porous membrane in non-deflected and deflected states, taken in the centre of the membrane. The threshold images were then used to calculate the centre to centre distance of the pores using Matlab. The calculated distances were then averaged to get a single value per image. Using the standard equation for strain, the linear strain inside the lung-on-chip was subsequently calculated. B: Shows the calculated linear strain values in the lung-on-a-chip array (n=12). C: Shows the differences between the linear strain (longitudinal strain), deflection, circumferential strain and surface strain created inside the lung-on-chip. The constant longitudinal and the varying circumferential strain sum up to a surface strain which depends on the location. Over all, the minimal surface strain the cells are experiencing inside the lung-on-chip is 8 % and the maximal is 16.6 %.

**Optical measurement technique to monitor the deflection of the thin, elastic and porous membrane**


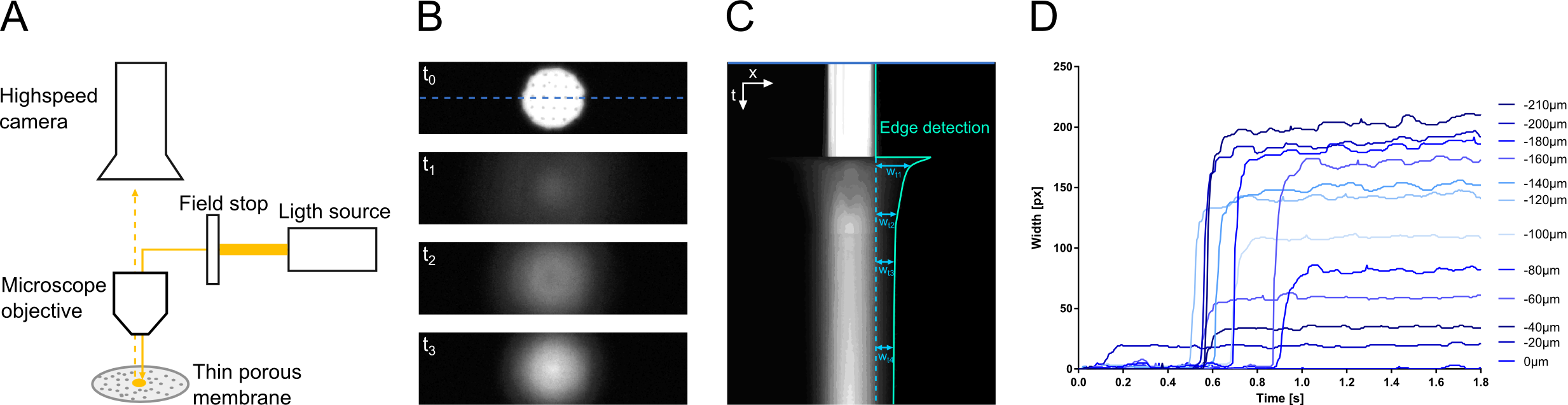


Supplementary Figure 4. Optical measurement technique to measure the fast deflection of the thin porous membrane. A: Measurement setup: The chip was placed on an upright microscope and a small light spot was projected on the membrane. The reflected light/ resulting image was then recorded by a high speed camera. During deflection, the shape of the projected light changes over time. B: Frames of the recorded images showing the shape change of the projected light spot over time. C: Example of a kymograph created form a recorded high-speed movie from the centre of the image (blue dashed line in B). From the kymograph the edges were then detected in Fiji (cyan coloured line) and the x- and y- values recorded. These values were then used to calculate the changes in width of the light spot (light blue) and thus the resulting deflection. D: Calibration curves used for the experiment. To obtain this curve, the membrane was deflected to a certain z-position and the deflection measured using the microscope. Then it was deflected to the same z-position while recording a high-speed movie. As a result, for each deflection (z-position) a different width was obtained. These data points were used to fit a calibration curve that was then used to correlate the measured width of the light spot to the corresponding deflection value.

**Shear stress**


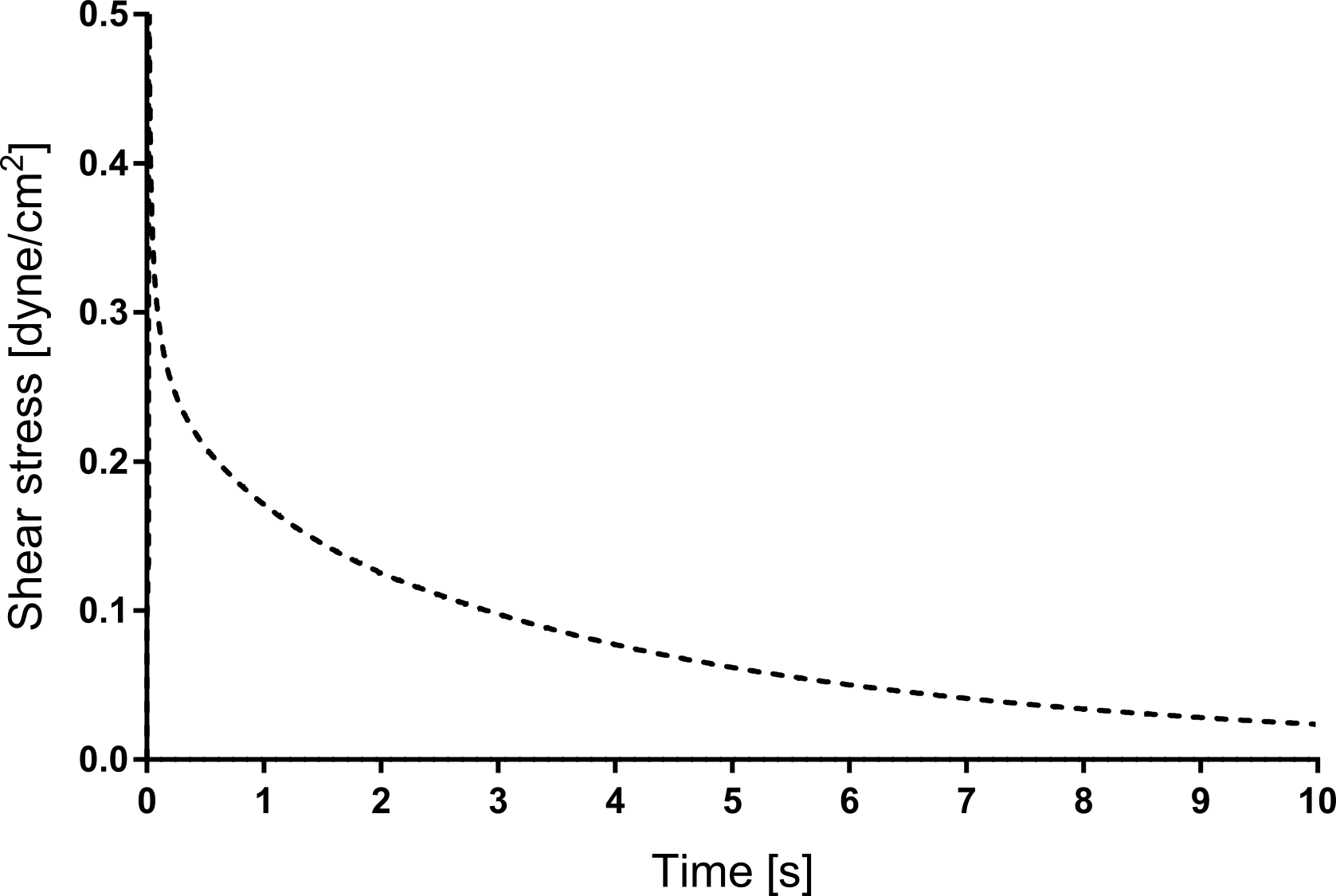


Supplementary Figure 5. Shear stress as a function of the time during medium exchange. The shear stress, *τ_w_*, was calculated using the following equation:

$$\tau_{w}=\frac{4\mu Q}{\pi R^{3}} , R=R_{hy}$$

With* *, *Q*, *R* and *R_hy_*, being the viscosity, the flow rate, the radius and the hydraulic radius, respectively. The maximal shear stress is about 5*10^-1^ dyne/cm^2^, if 150μL is filled in the inlet well. This value is smaller than the shear stress in human microvessels (3-10 dyne/cm^2^ [6]).

**TECAN reader compatibility**


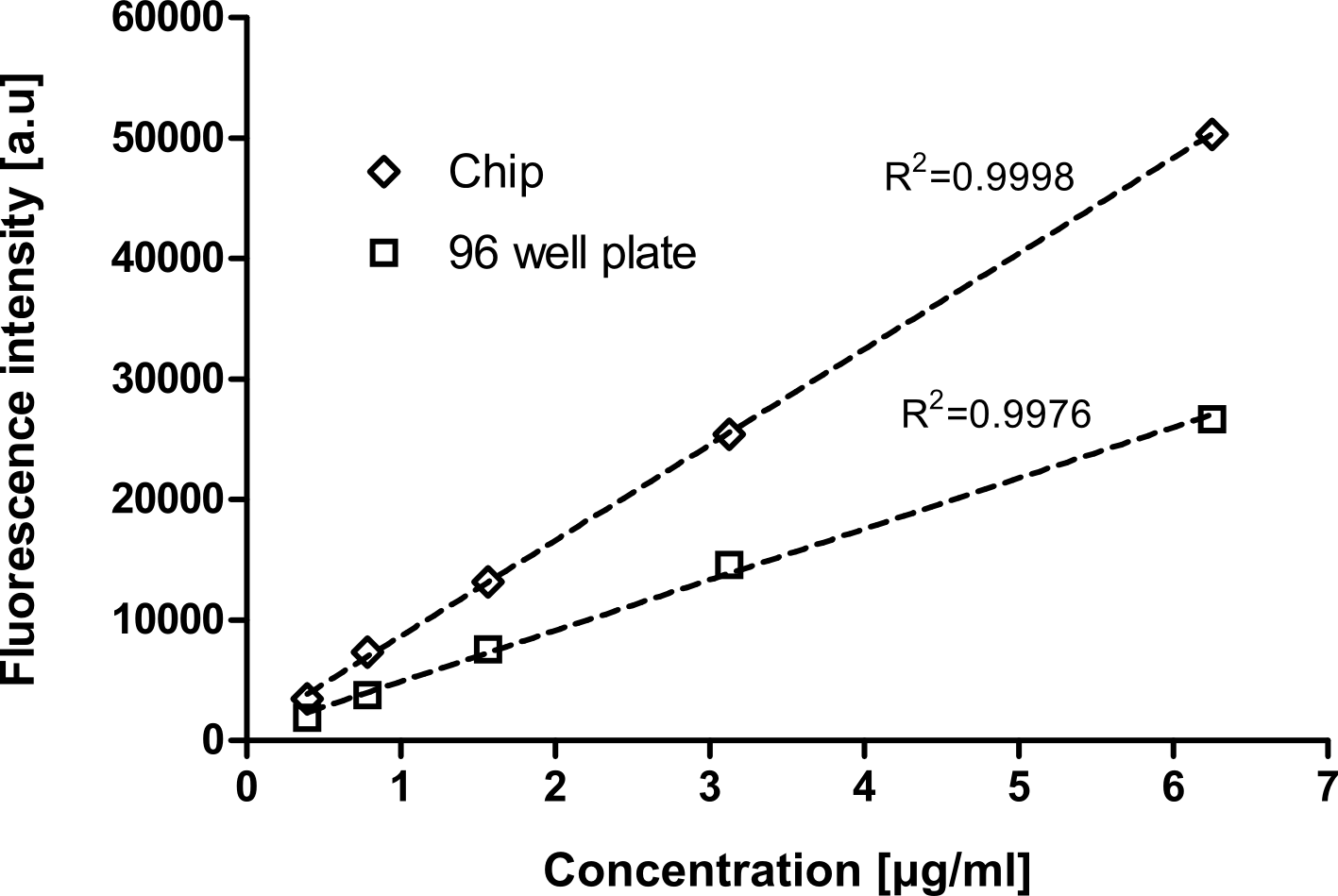


Supplementary Figure 6. Fluorescence intensity as function of the FITC solution concentration measured in a 96 well plate and inside the chip. Here it was investigated whether the chip can be used inside the TECAN reader as direct readout system. To do so different concentration of FITC were pipetted inside the apical well of the chip. Then the chip was placed inside a Nunc™ OmniTray™ plate and then inside the TECAN reader. As control the same amount of FITC solution was pipetted in a flat bottom well plate. These results confirm that fluorescence readouts can be directly made using the chip inside a TECAN reader.

**Characterisation of cell population**

**
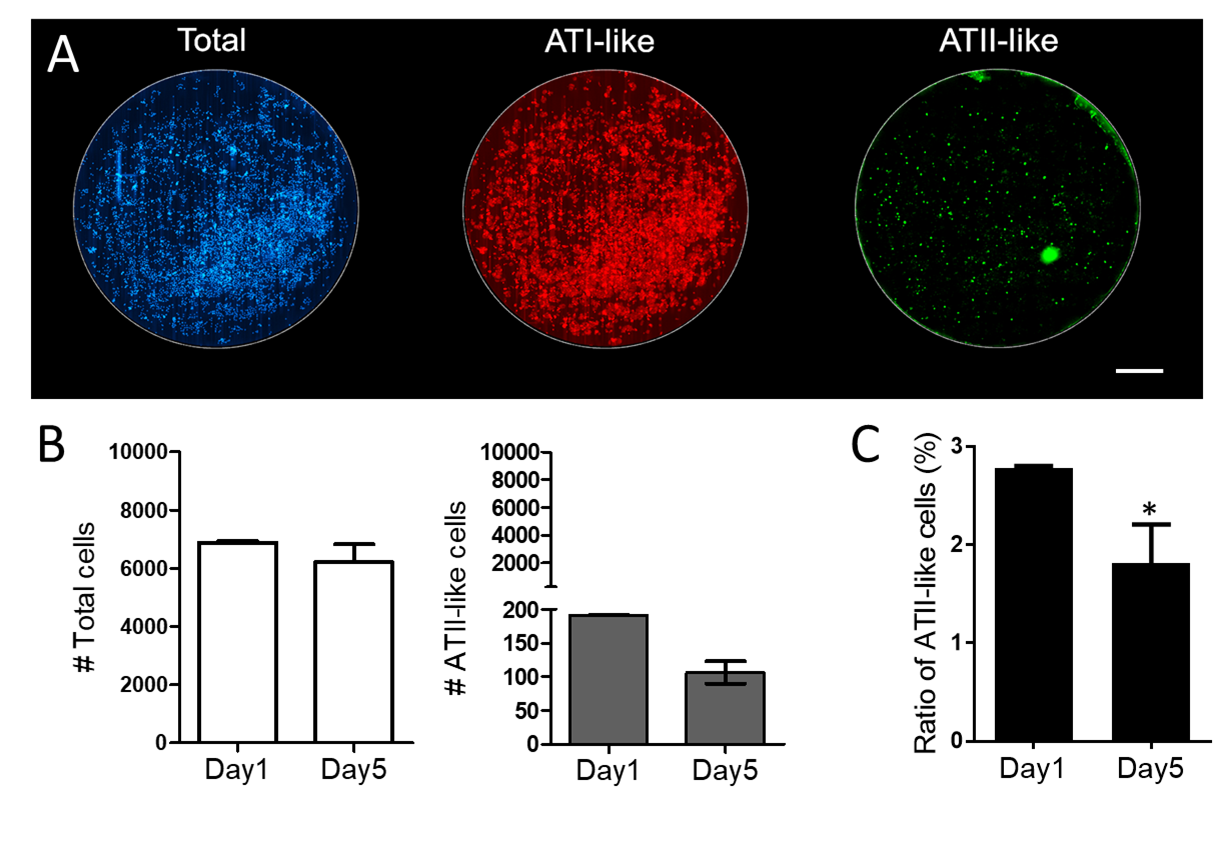
**

Supplementary Figure 7: Characterisation of the cell population on chip. A: Example of a whole chip screening. Image stitching was used to visualize the whole cell population in blue (DAPI), ATI-like cells in red (caveolin-1 positive) and ATII-like cells in green (ABCa3 positive) inside a well of the lung-on-chip. Scale bar: 500μm. B: Semi-automated read-outs were used to quantify the total number of cells and the number of ATII-like cells. The total number of cells was similar on day 1 and day 5. However, the number of ATII-like cells decreased from day 1 to day 5. C: Ratio of ATII-like cells to the whole cell population, demonstrating a decreasing fraction of ATII-like cells over time and thus confirming gene expression data.

**Characterisation of cell phenotype**


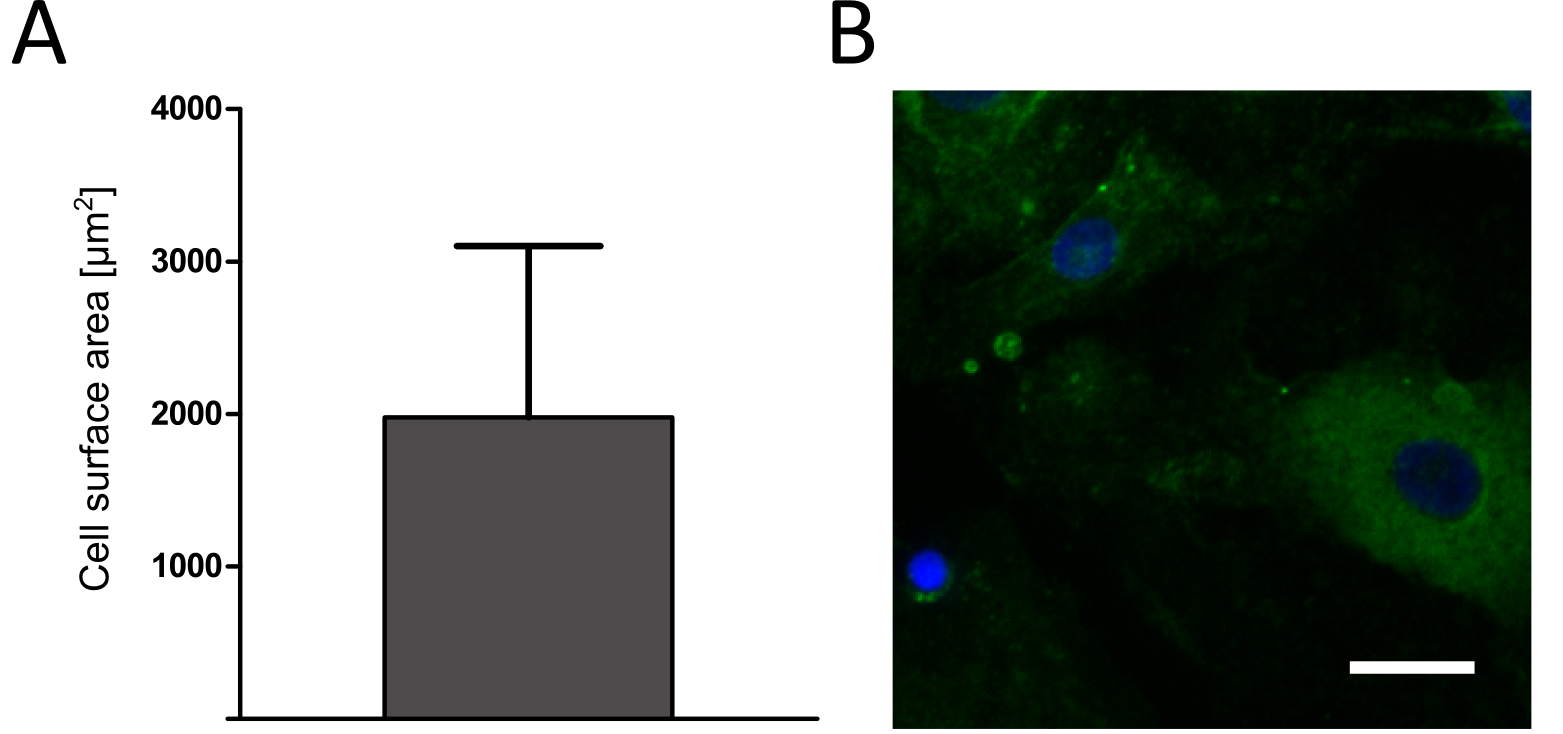


Supplementary Figure 8. Characterisation of cell phenotype on chip. A: Semi-automated read-outs were used to quantify the cell surface area using tight junction ZO-1 staining. B: Fluorescence micrograph showing the cell nuclei in blue and ATI-like cell stained with caveolin-1 in green.

Supplementary References

[1] W. Inman, K. Domansky, J. Serdy, B. Owens, D. Trumper, and L. G. Griffith, “Design, modeling and fabrication of a constant flow pneumatic micropump,” *J. Micromechanics Microengineering*, vol. 17, no. 5, pp. 891–899, May 2007.

[2] A. Flory, D. Brass, and K. Shull, “Deformation and adhesive contact of elastomeric membranes,” *J. Polym. Sci. Part …*, vol. 1, pp. 3361–3374, 2007.

[3] Fluxion, “Understanding effects of viscosity in the BioFlux system,” 2008. [Online]. Available: http://www.il-biosystems.de/fileadmin/Produkt-PDFs/168_BioFlux_Viscosity_TechNote-1038-01.pdf. [Accessed: 27-Jun-2016].

[4] Mediatech-Inc., “MATERIAL SAFETY DATA SHEET,” 2010. [Online]. Available: http://www.clearbridgebiomedics.com/pdf/Mediatech 1X PBS 21040.pdf. [Accessed: 27-Jun-2016].

[5] J. D. Michaels, J. E. Nowak, A. K. Mallik, K. Koczo, D. T. Wasan, and E. T. Papoutsakis, “Interfacial properties of cell culture media with cell-protecting additives,” *Biotechnol. Bioeng.*, vol. 47, no. 4, pp. 420–430, 1995.

[6] A. G. Koutsiaris, S. V Tachmitzi, N. Batis, M. G. Kotoula, C. H. Karabatsas, E. Tsironi, and D. Z. Chatzoulis, “Volume flow and wall shear stress quantification in the human conjunctival capillaries and post-capillary venules in vivo.,” *Biorheology*, vol. 44, no. 5–6, pp. 375–86, 2007.
